# Deficiency of TMEM53 causes a previously unknown sclerosing bone disorder by dysregulation of BMP-SMAD signaling

**DOI:** 10.1038/s41467-021-22340-8

**Published:** 2021-04-06

**Authors:** Long Guo, Aritoshi Iida, Gandham SriLakshmi Bhavani, Kalpana Gowrishankar, Zheng Wang, Jing-yi Xue, Juan Wang, Noriko Miyake, Naomichi Matsumoto, Takanori Hasegawa, Yusuke Iizuka, Masashi Matsuda, Tomoki Nakashima, Masaki Takechi, Sachiko Iseki, Shinsei Yambe, Gen Nishimura, Haruhiko Koseki, Chisa Shukunami, Katta M. Girisha, Shiro Ikegawa

**Affiliations:** 1 Laboratory for Bone and Joint Diseases, RIKEN Center for Integrative Medical Sciences, Tokyo, Japan; 2 Department of Clinical Genome Analysis, Medical Genome Center, National Center of Neurology and Psychiatry, Kodaira, Tokyo, Japan; 3 Department of Medical Genetics, Kasturba Medical College, Manipal, Manipal Academy of Higher Education, Manipal, India; 4 Kanchi Kamakoti CHILDS Trust Hospital, Tamil Nadu, India; 5 Department of Medical Genetics, Institute of Basic Medical Sciences, Peking Union Medical College and Chinese Academy of Medical Sciences, Beijing, China; 6 Department of Human Genetics, Yokohama City University Graduate School of Medicine, Yokohama, Japan; 7 Department of Ultrasound, The Second Affiliated Hospital, Medical School of Xi’an Jiaotong University, Xi’an, China; 8 Laboratory for Developmental Genetics, RIKEN Center for Integrative Medical Sciences, Yokohama, Japan; 9 Department of Cell Signaling, Graduate School of Medical and Dental Sciences, Tokyo Medical and Dental University, Tokyo, Japan; 10 Department of Molecular Craniofacial Embryology, Graduate School of Medical and Dental Sciences, Tokyo Medical and Dental University, Tokyo, Japan; 11 Department of Molecular Biology and Biochemistry, Graduate School of Biomedical and Health Sciences, Hiroshima University, Hiroshima, Japan

**Keywords:** Disease genetics, Development, Next-generation sequencing, Growth disorders

## Abstract

Bone formation represents a heritable trait regulated by many signals and complex mechanisms. Its abnormalities manifest themselves in various diseases, including sclerosing bone disorder (SBD). Exploration of genes that cause SBD has significantly improved our understanding of the mechanisms that regulate bone formation. Here, we discover a previously unknown type of SBD in four independent families caused by bi-allelic loss-of-function pathogenic variants in *TMEM53*, which encodes a nuclear envelope transmembrane protein. *Tmem53*^*-/-*^ mice recapitulate the human skeletal phenotypes. Analyses of the molecular pathophysiology using the primary cells from the *Tmem53*^*-/-*^ mice and the *TMEM53* knock-out cell lines indicates that TMEM53 inhibits BMP signaling in osteoblast lineage cells by blocking cytoplasm-nucleus translocation of BMP2-activated Smad proteins. Pathogenic variants in the patients impair the TMEM53-mediated blocking effect, thus leading to overactivated BMP signaling that promotes bone formation and contributes to the SBD phenotype. Our results establish a previously unreported SBD entity (craniotubular dysplasia, Ikegawa type) and contribute to a better understanding of the regulation of BMP signaling and bone formation.

## Abstract (web summary)

Sclerosing bone disorder (SBD) includes a broad spectrum of monogenic diseases characterised by increased bone density. Here, the authors describe a previously unknown SBD in four families caused by mutations in TMEM53 and demonstrate the role this protein plays in BMP signalling during bone formation.

## Introduction

Sclerosing bone disorder (SBD) is a heterogeneous group of monogenic diseases characterized by increased bone density. More than 40 disease entities, including osteopetrosis, dysosteosclerosis, and sclerosteosis, fall into this category^1^, which encompasses a broad phenotypic spectrum involving lethality, bone fracture risk, and complications. Identification of dozens of the causal genes for SBD over the past few decades has not only improved precise diagnosis and genetic counseling of the diseases but also continuously driven the research of molecular mechanisms of bone formation and regulation of the skeletal system and exploration of innovative therapies for bone density-related diseases^2–5^.

The bone morphogenetic protein (BMP) signaling pathway plays important roles in bone formation^6^ and is related to several human skeletal dysplasias^7–9^. Binding of BMP2/4 to their receptors initiates the signal transduction cascade by inducing phosphorylation of SMAD1/5/9, which can then form hetero-complexes with SMAD4 followed by translocation into the nucleus to upregulate osteogenesis-related genes. However, the mechanism by which nuclear translocation is regulated remains unclear.

In this study, we discover a previously unknown type of SBD and identify its causal gene, *TMEM53*, which encodes nuclear envelope transmembrane (NET) protein 53 (TMEM53, also known as NET4). TMEM53 is initially identified in a screen for nuclear envelope proteins^10^ and then later confirmed to localize to the outer membrane of the nucleus^11^. Its function remains unclear, particularly in bone formation. We show that TMEM53 acts as an inhibitor of BMP-SMAD signaling by preventing SMAD accumulation in the cell nucleus and that its deficiency enhances osteogenic differentiation by overactivating the BMP signaling pathway. Our findings establish a previously unreported SBD entity and demonstrate a regulatory mechanism in the BMP signaling pathway that impacts bone formation and development.

## Results

### Identification of *TMEM53* pathogenic variants in a previously unknown type of SBD

**Fig. 1:**
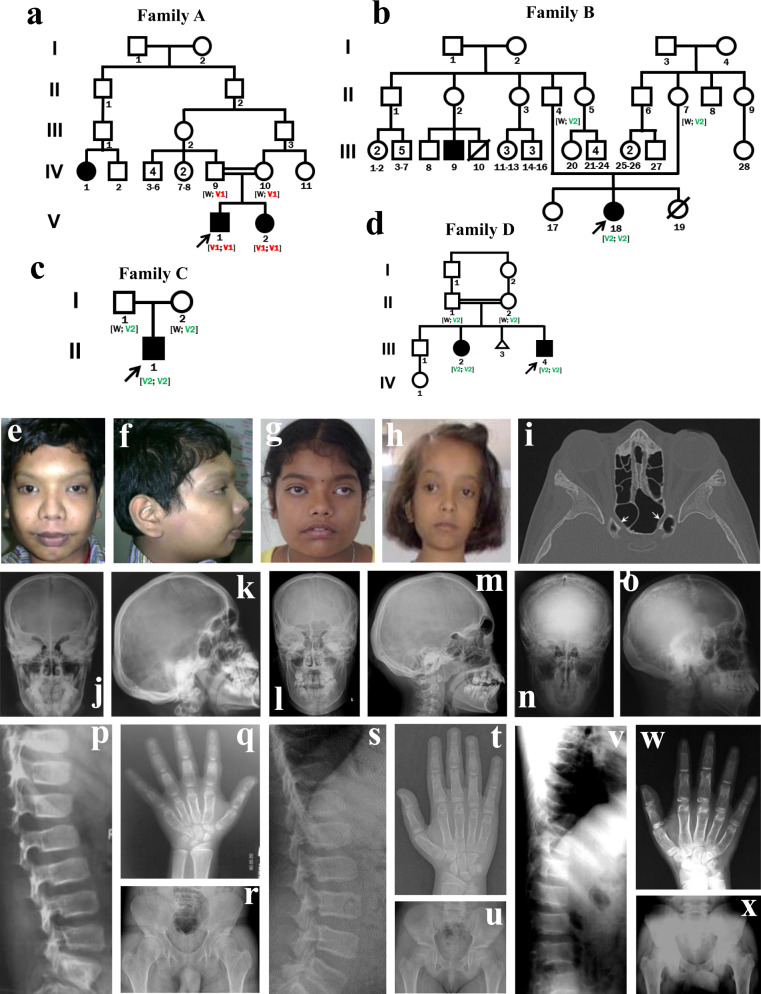
A previously unknown type of skeletal dysplasia associated with *TMEM53* variants. **a**–**d** Pedigrees of Families A–D. A previously unknown skeletal dysplasia co-segregated with two recessive *TMEM53* variants, c.222_223insCATG (V1) and c.62-5_62-3delTTC (V2), in four independent families. Black filled symbols indicate affected individuals; black arrows indicate probands. **e**–**h** Physical appearance of the patients. Prominent forehead and glabella, dolichocephaly, broad nasal root, hypertelorism, and strabismus in individual A-V-1 (**e**, **f**), A-V-2 (**g**), and B-III-18 (**h**). **i** CT image shows narrowing of the optic canal (arrow) in individual A-V-2. **j**–**x** Radiological features of individual A-V-1 (**j**, **k**, **p**, **q**, **r**), individual A-V-2 (**l**, **m**, **s**, **t**, **u**), and individual C-II-1 (**n**, **o**, **v**, **w**, **x**). **j**–**o** Skull X-ray images show diffuse thickening of calvaria, minor sclerosis of skull base, and prominent frontal bones. **p**, **s**, **v** Lateral views of the spine displays mild platyspondyly and broad ribs. **q**, **t**, **w** Hand X-ray images reveal mild brachydactyly and under-constriction of diaphyses of metacarpals and phalanges. **r**, **u**, **x** Pelvis X-ray images show widening of pubis and ischia, broadening of the femoral neck, and meta-diaphyseal under-modeling.

We recruited individuals with SBD from the Japanese Skeletal Dysplasia Consortium (http://www2.riken.jp/lab/OA-team/JSDC/). Detailed clinical analyses identified five individuals in four independent Indian families who were affected by the same unknown type of SBD (Fig. 1a–d); the families resided in different geographic areas of India. Pre-natal and early post-natal development were uneventful, but all five showed proportional or short-limbed short stature with variable severity, which was not identifiable at birth (Table 1). In addition, the patients had various kinds of head deformities (macrocephaly, dolichocephaly, or prominent forehead), and epicanthic folds and thick vermilion of upper and lower lips were common. Their vision diminished progressively after early childhood due to optic nerve compression, and neither intellectual disability, dental problems, nor chest or spinal deformities were present. Their radiographic features were hyperostosis of the calvaria and the skull base, mild platyspondyly, wide pubis and ischia, broadening of the femoral neck, meta-diaphyseal under-modeling of the long tubular bones, and mild shortening and diaphyseal broadening of the short tubular bones (Table 1 and Fig. 1e–x), indicating that the disorder belongs to the group of craniotubular dysplasias. Laboratory findings including calcium, phosphorus, parathyroid hormone, and alkaline phosphatase (ALP) were unremarkable. A search of known skeletal disorders, including disease entities registered in the latest version of the international nosology and classification of genetic skeletal disorders^1^, did not find any reports matching the characteristic skeletal phenotypes that were observed in the five patients.

**Table 1:** Clinical and radiographic findings of the four Indian families with homozygous *TMEM53* pathogenic variants.

Individual	V-1	V-2	III-18	II-1	III-4
Family	A	A	B	C	D
Pathogenic variant	c.222_223ins4	c.222_223ins4	c.62-5_c.62-3del3	c.62-5_c.62-3del3	c.62-5_c.62-3del3
Demographics					
Sex	Male	Female	Female	Male	Male
Present age	19 years	12 years	8 years	15 years	17 years
Consanguinity	+	+	Probably +	−	+
Clinical finding					
Height [cm] (SD)	150 (−3.6)	138 (−2.7)	107 (−3.6)	148 (−3)	138 (−5)
Macrocephaly	−	−	+	+	+
Prominent forehead	+	+	+	N/A	N/A
Hypertelorism	+	+	+	−	+
Dolichocephaly	+	+	+	−	+
Impaired vision	Started at 8 years	Started at 11 years	Started at 8 years	Started at 11 years	Started at 17 years
Radiographic finding					
Thin cortex	+	+	+ Mild	+ Mild	−
Calvaria thickening	+	+	+ Mild	+	+
Skull base sclerosis	+	+ Mild	−	+	+
Broad rib	+	+	+ Mild	+	+ Mild
Platyspondyly	+ Mild	+ Mild	+ Mild	+ Mild	+ Mild
Wide pubis/ischia	+	+	+ Mild	+	+
Broad femoral neck	+	+	+	+	+
Meta-diaphyseal undermodeling of long bones	+	+	+ Mild	+	+ Very mild
Broadening diaphysis of short bones	+	+	+	+	+ Very mild

*N/A* not available.

**Fig. 2:**
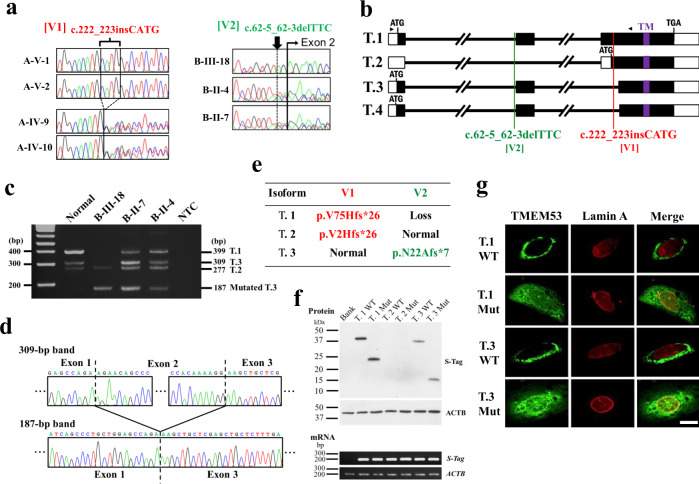
Functional analysis of *TMEM53* pathogenic variants. **a** Electropherograms of Sanger sequencing showing that two variants in *TMEM53*, c.222_223insCATG (V1) and c.62-5_62-3delTTC (V2), were homozygous in the affected individuals in Families A and B, respectively. The unaffected parents were heterozygotes for these variants. **b** The location of the two variants in the four RefSeq transcripts of *TMEM53*. T.1, NM_024587.4; T.2, NM_001300746.1; T.3, NM_001300747.2; T.4, NM_001300748.2; TM, transmembrane domain. The arrow heads represent the primers used in RT-PCR of **c**. **c** RT-PCR analysis for the peripheral blood from the affected individual (B-III-18) and the unaffected parents (B-II-4 and B-II-7) (*n* = 2 independent experiments). Compared to the normal control, an extra 187-bp band was detected in B-III-18, B-II-4, and B-II-7. The 399-bp and 309-bp bands corresponding to T.1 and T.3, respectively, were not detected in B-III-18. T.4 with three nucleotides less than T.3 was not detected in any individuals. NTC, no template control. **d** Electropherograms of Sanger sequencing showing that the 187-bp RT-PCR band represents a T.3 variant, in which exon 2 is skipped. The variant T.3 contains a premature stop codon in exon 3, resulting in a truncated protein p.N22Afs*7. **e** The effects of the two variants at protein levels. V1 causes a frameshift in transcripts T.1 and T.2, thus leading to two truncated proteins, p.V75Hfs*26 and p.V2Hfs*26, respectively. V2 causes T.1 loss and T.3 aberrant splice producing p.N22Afs*7. **f** Forced expression of wild-type (WT) and truncated TMEM53 proteins with S-tag in MG-63 cells (*n* = 2 independent experiments). T.1 Mut, p.V75Hfs*26; T.2 Mut, p.V2Hfs*26; T.3 Mut, p.N22Afs*7. T.2 WT and T.2 Mut were transcribed but not translated. **g** Immunofluorescence staining for S-tag-TMEM53 and Lamin A in MG-63 cell (*n* = 3 independent experiments). T.1 Mut and T.3 Mut failed in nuclear envelope targeting, while T.1 WT and T.3 WT were expressed around the nuclear envelopes. The scale bar represents 20 μm. Source data are provided as a Source Data file.

With the pedigrees indicating a likely autosomal recessive mode (Fig. 1a–d), we performed whole-exome sequencing (WES) (Supplementary Table 1) and identified two homozygous variants, c.222_223insCATG (ClinVar, SCV001446315) in Family A and c.62-5_62-3delTTC (ClinVar, SCV001446316) in Families B–D, in *TMEM53* (Fig. 1a–d) that were not present in any known population genetic databases including gnomAD and ExAC. We confirmed the variants by Sanger sequencing of genomic DNA from the affected subjects and their parents (Fig. 2a) and found that the variants co-segregated with the disease in the families. Homozygosity mapping using the WES data indicated that individuals B-III-18, C-II-1, and D-III-4 shared a 2.4-Mb homozygous region including *TMEM53* (Supplementary Fig. 1), suggesting a common ancestral origin for c.62-5_62-3delTTC.

*TMEM53* has four RefSeq transcripts, here named T.1 (NM_024587.4), T.2 (NM_001300746.1), T.3 (NM_001300747.2), and T.4 (NM_001300748.2), with T.1 showing the highest expression in multiple tissue and cell types (Supplementary Fig. 2). The variant c.222_223insCATG is located in the coding sequences of T.1 and T.2 (Fig. 2b), thus causing a frame-shift in exon 3. Because exon 3 is the last exon, nonsense-mediated mRNA decay (NMD) would not occur. Instead, the mutated T.1 and T.2 would produce two truncated proteins, p.V75Hfs*26 and p.V2Hfs*26 (NP_078863.2), respectively, that lack the transmembrane domain (Supplementary Fig. 3a).

The variant c.62-5_62-3delTTC neighbors the intron 1–exon 2 junction (Fig. 2b) and is predicted to cause abnormal splicing by multiple online programs (Supplementary Table 2). RT-PCR analysis using RNA extracted from the peripheral blood of one affected individual (B-III-18) and their unaffected parents (B-II-4 and B-II-7) who carry c.62-5_62-3delTTC indicated an extra short band (Fig. 2c), which corresponds to a T.3 variant in which exon 2 is skipped during the splicing process (Fig. 2d and Supplementary Fig. 3b). As a result, a premature stop codon is generated in exon 3, resulting in a truncated protein, p.N22Afs*7　(NP_078863.2), which also lacks a transmembrane domain (Supplementary Fig. 3b). Unlike the complete loss of normal T.3 in the affected individual, normal T.3 was still detected in the heterozygous parents (Fig. 2c). Notably, the band corresponding to T.1 in the normal individual and heterozygous parents did not exist in the affected individual (Fig. 2c), indicating that c.62-5_62-3delTTC leads to complete loss of the T.1 transcript, which has both the highest expression in normal individuals and represents the longest protein coding sequence.

In summary, c.222_223insCATG in Family A potentially generates two truncated proteins (p.V75Hfs*26 and p.V2Hfs*26), and c.62-5_62-3delTTC in Families B–D causes T.1 loss and generates one truncated protein, p.N22Afs*7 (Fig. 2e). All three truncated proteins lack the transmembrane domain, which is necessary for nuclear envelope targeting of transmembrane proteins^10, 11^. To assess their subcellular localization, we expressed these truncated proteins and the corresponding normal isoforms with S-tag in the MG-63 cell line (Fig. 2f). Immunocytochemistry (ICC) for the tagged proteins indicated that p.V75Hfs*26 and p.N22Afs*7 failed to target the nuclear envelopes, where their normal isoforms were localized (Fig. 2g). T.2 and its truncated protein, p.V2Hfs*26, were expressed on mRNA, but not on protein level (Fig. 2f), which is consistent with the observation that the vast majority of genes have a single dominant splice isoform that is translated regardless of the variety of alternative splice variants predicted to exist at the transcript level^12^. Since T.1 shows the highest expression in multiple tissue types, including cartilage (Supplementary Fig. 2), and its disruption is a sufficient condition to cause the disease in the patients with c.222_223insCATG (Fig. 2e), T.1 is probably the most important transcript in the developing skeleton.

### *Tmem53* mutant mice recapitulated the human SBD associated with *TMEM53* pathogenic variants

TMEM53 is highly conserved among species and is 86.3% identical between human and mouse (Supplementary Fig. 4). To generate *Tmem53*-deficient mice, we introduced deleterious mutations into the coding region by CRISPR/Cas9-mediated gene editing. A targeting site shared by all six RefSeq transcripts of *Tmem53* was selected to guarantee that the mutation could disrupt all transcripts (Supplementary Fig. 5a). As a result, three lines with three frame-shift mutations that would produce truncated proteins without a transmembrane domain in all transcripts were established (Supplementary Fig. 5b, c), and all of the lines showed late-onset short stature. Phenotypes of the line *Tmem53*^*1bin/1bin*^ were evaluated in detail in the following experiments.

**Fig. 3:**
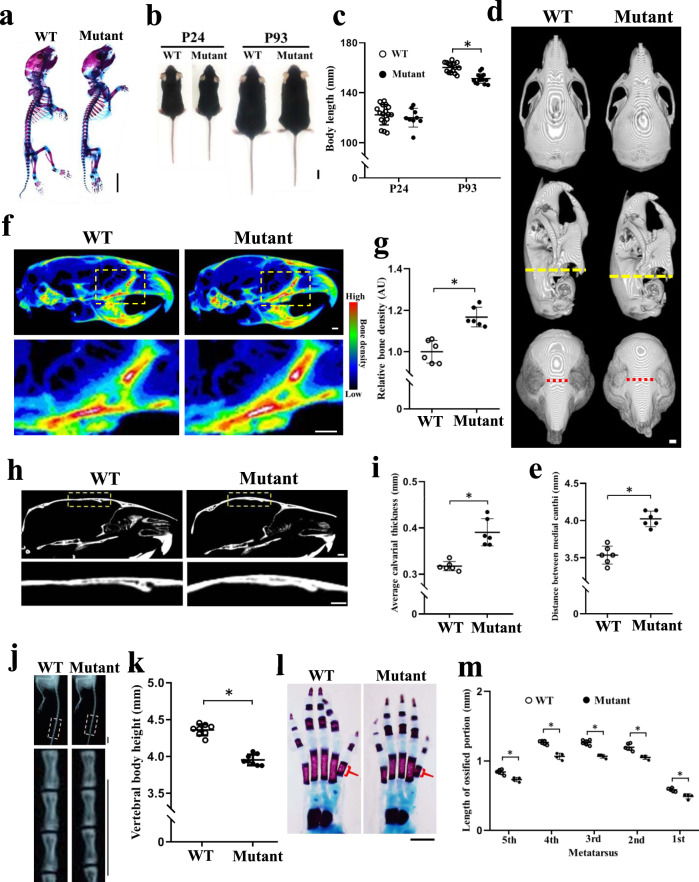
*Tmem53* mutant mice recapitulate the human skeletal dysplasia associated with *TMEM53* pathogenic variants. **a** Lateral views of skeletal preparation of wild-type (WT) and *Tmem53* mutant mice (Mutant) at P7. Scale bar, 5 mm. **b** Dorsal views of male WT and Mutant at P24 and P93. Scale bar, 1 cm. **c** Measurement of the body length (*n* = 9, 15, 15, 17). *P* = 0.48 and 1 × 10^−6^* for P24 and P93, respectively. **d** Representative 3D images reconstructed from micro-CT stacks of P113 mouse skull. The upper, middle, and lower panels show dorsal, lateral, and frontal views, respectively. The yellow and red dotted lines show the skull height and the distance between the medial canthi of bony orbits, respectively. Scale bar, 1 mm. **e** Measurement of the distance between medial canthi (*n* = 6). *P = 0.000021. **f** Lateral views of color-coded Z-projection images of Micro-CT stacks. The areas highlighted by the dotted box in the upper panels are magnified in the lower panels. Scale bar, 1 mm. **g** Quantification of relative bone density of the skull bases (*n* = 6). AU, arbitrary unit. *P = 0.0002. **h** Sagittal micro-CT images of adult mice. The lower panels are the areas highlighted in the upper panels. Scale bar, 1 mm. **i** Quantification of the calvarial thickness (*n* = 6). *P = 0.0002. **j** Lateral views of X-ray images of P113 mouse tails. The areas highlighted by the dotted box in the upper panels are magnified in the lower panels. Scale bar, 1 cm. **k** Measurement of the average height of the three caudal vertebral bodies shown in **j** (*n* = 8). *P = 0.00000003. **l** Skeletal preparation of left hind paws at P7. The red bracket shows the ossified portion of the first metatarsus. Scale bar, 1 mm. **m** Quantitative analysis of the ossified portion of the metatarsal bones (*n* = 4 and 6). *FDR-adjusted *P* = 0.0010, 0.000051, 0.000051, 0.00088, and 0.0025. The above quantitative data indicate mean ± SD and statistical significance was assessed using two-sided *t*-test. Source data are provided as a Source Data file.

As summarized in Table 1, all affected individuals who were homozygous for *TMEM53* pathogenic variants showed normal development until birth and late-onset short stature. Similarly, the *Tmem53* mutant mice did not display severe skeletal abnormity at birth (Fig. 3a) but later exhibited late-onset short stature (Fig. 3b, c). The craniofacial dysmorphias observed in the affected subjects, including tall forehead (Fig. 1f, k, m, o) and hypertelorism (Fig. 1e, g, h), were also found in the *Tmem53* mutant mice (Fig. 3d, e and Supplementary Fig. 6). In addition, the mutant mice showed thickening of the calvaria and minor sclerosis of the skull base (Fig. 3f–i), which we highlighted for the affected individuals (Fig. 1k, m, o). Like some other SBDs^5^, the cranial nerve compression caused by bony overgrowth of the cranial nerve foramina also existed in the affected individuals, who had optic nerve compression with resultant vision impairment (Fig. 1i and Table 1). Although the vision of the *Tmem53* mutant mice was not evaluated in this study, hyperostosis of the skull base (Fig. 3f) and narrowing of the bony fissure relevant to the optic foramen were observed in the mice (Supplementary Fig. 7). Another radiographic feature of the SBD was platyspondyly, i.e., flattened vertebral bodies throughout the axial skeleton (Fig. 1p, s, v), which was also identified in the *Tmem53* mutant mice (Fig. 3j, k). With respect to the tubular bones, the patients showed proportionally short limbs and hands, and under-constriction of the diaphyses (Fig. 1q, t, w), which were also observed in the mutant mice (Fig. 3l–m and Supplementary Fig. 8). Thus, the *Tmem53* mutant mice recapitulated the skeletal features identified in the affected subjects, who were homozygous for *TMEM53* pathogenic variants, reinforcing the genetics-based hypothesis presented above that *TMEM53* is the causal gene for the previously unknown type of SBD.

### Enhanced osteoblast differentiation in the calvaria of the *Tmem53* mutant mice

**Fig. 4:**
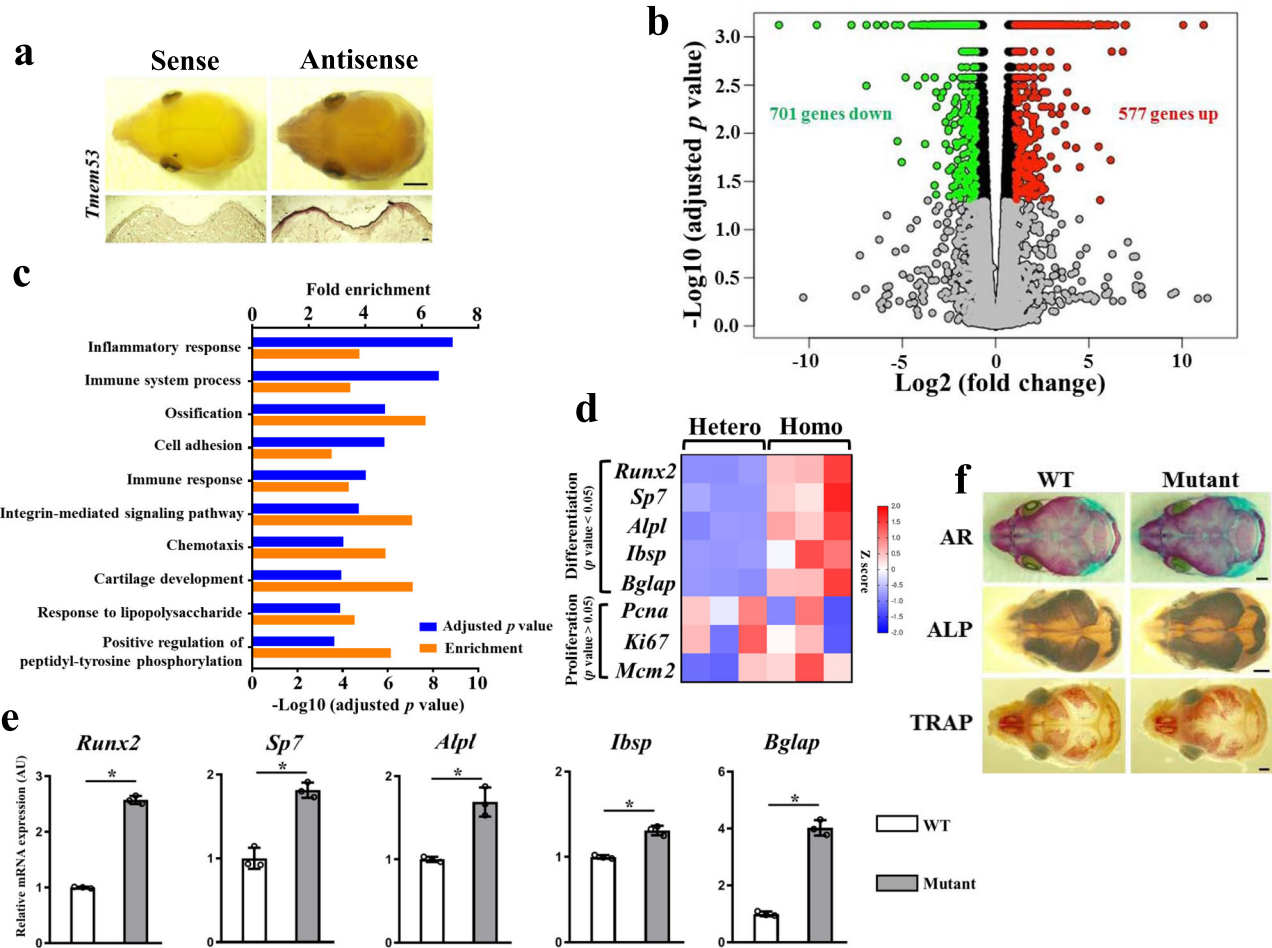
Enhanced differentiation of calvaria osteoblast in *Tmem53* mutant mice. **a** In situ hybridization (ISH) for *Tmem53*. The upper panels show dorsal views of cephalic whole-mount ISH at E16.5, scale bar, 1 mm; the lower panels show coronal ISH sections of the calvaria at P1, scale bar, 100 μm (*n* = 3 mice). **b** Volcano plot of RNA-seq data for calvaria showing differentially expressed genes (DEGs) in *Tmem53* mutant mice (Homo) versus their heterozygous littermates (Hetero) (*n* = 3 animals for each group) with respect to fold change (FC) and significance (adjusted *P* value). Each dot represents an individual gene. Gray dots represent genes with no significantly differentially expressed (*P* > 0.05). Green dots represent significant downregulated DEGs (FC < 0.5) and red dots represent significant upregulated DEGs (FC > 2). Statistical significance was assessed using two-sided *t*-test. *P* value is adjusted by false discovery rate (FDR). **c** Gene ontology (GO) enrichment analysis for the DEGs by DAVID 6.8 showing top 10 significantly enriched GO terms for the DEGs in biological processes. Statistical significance was assessed using one-sided Fisher’s exact probability test. *P* value is adjusted by FDR. **d** Heat map of representative DEGs associated with osteoblast differentiation and representative genes associated with cell proliferation. **e** RT–qPCR-based validation of the DEGs found via RNA-seq to be upregulated in the calvaria of *Tmem53* mutant mice. WT, wild type. Data are mean ± SD (*n* = 3 biologically independent samples). *P = 0.000003, 0.0008, 0.0025, 0.0008, and 0.00005 for *Runx2*, *Sp7*, *Alpl*, *Ibsp*, and *Bglap*, respectively. Statistical significance was assessed using two-sided *t*-test. **f** Dorsal views of the skull stained by Alizarin Red (AR) at P2, alkaline phosphatase staining (ALP) at E16.5, and tartrate-resistant acid phosphatase (TRAP) at P4, respectively (*n* = 3 animals for each group). Scale bar, 1 mm. Source data are provided as a Source Data file.

To identify the molecular mechanisms underlying the skeletal phenotypes caused by TMEM53 deficiency, we examined the expression pattern of *Tmem53* in mouse heads. In situ hybridization for *Tmem53* showed high expression in calvaria (Fig. 4a), which were thickened in the *Tmem53* mutant mice (Fig. 2h) and the *TMEM53* mutant individuals (Fig. 1k, m, o). Then, the calvaria dissected from the *Tmem53* mutant mice and its heterozygous siblings were subjected to RNA sequencing (RNA-seq). From a total of 14,836 genes on which gene expression profiling was performed, we identified 1278 differentially expressed genes (DEGs) (Fig. 4b). These DEGs were enriched for some Gene Ontology (GO) terms, and the most enriched term was “ossification” (Fig. 4c), which can be considered as closely related to thickening of the calvaria in the *Tmem53* mutants, since a calvarium is formed via intramembranous ossification, in which bone develops directly from sheets of undifferentiated mesenchymal connective tissue^13^. To assess the bone differentiation status of the calvaria, we examined the known marker genes of osteogenic precursors, osteoblasts, and osteocytes in the RNA-seq dataset (Fig. 4d) and validated the findings by RT–qPCR (Fig. 4e). Osteogenic precursor and early osteoblast markers *Runx2* and *Sp*7 were increased (Fig. 4d, e), suggesting enhanced differentiation of mesenchymal stem cells into pre-osteoblasts in the mutant calvaria. Accordingly, the downstream genes of *Runx2* and *Sp7*, *Alpl*, *Bglap* and *Ibsp*, were upregulated as well (Fig. 4d, e). The elevated genes are late osteoblast and osteocyte markers, thus revealing increased bone formation in the *Tmem53* mutant mice, which was further confirmed by ALP and Alizarin Red (AR) staining of the wild-type and mutant calvaria (Fig. 4f). We also checked the proliferation markers in the RNA-seq dataset, and no significant change was recorded (Fig. 4d). A methyl thiazolyl tetrazolium (MTT) assay for the primary calvarial cells did not show a difference for cell growth (Supplementary Fig. 9). These results support the hypothesis that the calvaria thickening in the *Tmem53* mutant is caused by enhanced osteoblast differentiation rather than proliferation.

### TMEM53 deficiency overactivates the BMP signaling pathway during calvaria ossification

**Fig. 5:**
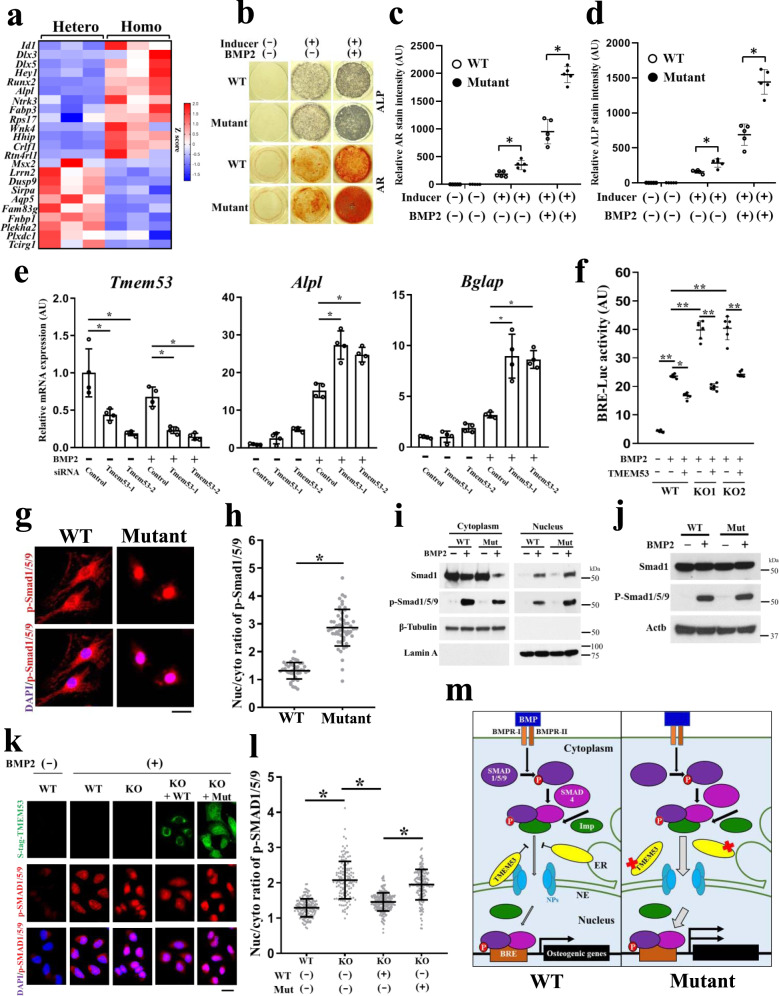
TMEM53 deficiency upregulates BMP-SMAD signaling by facilitating p-SMAD1/5/9 nuclear translocation. **a** Heat map of representative Smad1/5/9 target genes expressed differentially between *Tmem53* mutant mice (Homo) and their heterozygous littermates (Hetero). **b** Alkaline phosphatase (ALP) and Alizarin Red (AR) staining of primary calvaria cells. **c**, **d** Quantification of ALP and AR staining; *n* = 5 biologically independent samples. FDR-adjusted *P* = 0.60, 0.011*, and 0.000073* for AR staining. *P* = 0.18, 0.0012*, and 0.000089* for AR staining. AU, arbitrary unit. **e** qPCR for BMP2-induced osteoblast markers in MC3TC-E1 cells; *n* = 4 biologically independent samples. *P < 1 × 10^−5^. **f** Smad1/5/9-mediated BMP2 signaling activity in Hela cells with *TMEM53* depletion or overexpression. KO1 and KO2: two *TMEM53* knockout lines. BRE, BMP response element; *n* = 6 biologically independent samples. *P = 9.3 × 10^−11^, **P < 1 × 10^−15^. **g** Immunocytochemistry (ICC) for P-Smad1/5/9 in BMP2-treated primary calvaria cells. Scale bar, 20 μm. **h** Quantification of nuclear to cytoplasmic (nuc/cyto) ratio of P-Smad1/5/9 in **g**; 42 and 54 cells were quantified, *P < 1 × 10^−15^. **i** Western blot of cytoplasmic and nuclear fractions of WT and mutant (Mut) primary calvaria cells; *n* = 3 independent experiments. **j** Western blot of whole-cell lysate; *n* = 3 independent experiments. **k** ICC of BMP2-treated WT and *TMEM53* knockout (KO) Hela cells. WT and p.V75Hfs*26 (Mut) TMEM53 were expressed in the KO cells to rescue the KO effect. Scale bar, 20 μm. **l** Quantification of nuc/cyto ratio of P-SMAD1/5/9 in **k**; 149, 140, 237, and 149 cells were quantified. *P < 1 ×10^-15^. The data indicate mean ± SD. Statistical test: two-sided *t*-test for **c**, **d**, and **h**; one-way ANOVA for **e**, **f**, and **l**. **m** Working model. TMEM53 works as a gate-keeper of BMP-SMAD signal at outer nuclear membranes to inhibit the BMP-activated osteogenic pathway. The *TMEM53* pathogenic variants cause loss-of-function of TMEM53, thus facilitating the nuclear accumulation of P-Smad1/5/9. Imp, importins; NPs, nucleoporins; BRE, BMP response element; NE, nuclear envelope; ER, endoplasmic reticulum. Source data are provided as a Source Data file.

The BMP signaling pathway plays important roles in the craniofacial development by upregulating the osteogenesis-related genes such as *Dlx5* and *Runx2* (refs. ^14–16^). Since Runx2 and other downstream markers of bone formation were increased in the calvaria of *Tmem53* mutant mice (Fig. 4d, e), we hypothesized that *Tmem53* deficiency could enhance osteoblast differentiation by modulating the BMP signaling pathway. To verify the hypothesis, we performed an enrichment analysis using the calvaria RNA-seq dataset and found that the DEGs were enriched for the reported target genes of Smad1/5/4 (ref. ^17^) (Supplementary Fig. 10). Notably, the representative downstream transcriptional targets of BMP signaling, *Id1*, *Dlx3*, and *Dlx5* (refs. ^18–20^), were significantly upregulated in the *Tmem53* mutant (Fig. 5a), suggesting overactivated BMP-SMAD signaling in the calvaria.

We then assessed the bone formation capacity of the primary calvaria cells by in vitro induction. The ALP and AR staining showed increased osteoblastic activity and calcification in *Tmem53* mutant cells upon induction of osteoblast differentiation (Fig. 5b–d). Notably, the difference of bone formation capacity between wild-type and mutant calvaria cells was increased by BMP2 stimulation (Fig. 5b–d) and ablated by adding K02288, a selective inhibitor of the BMP type I receptor kinases^21^ (Supplementary Fig. 11). We further evaluated markers of bone formation in response to BMP2 stimulus using MC3T3-E1, a mouse calvaria-derived osteoblastic cell line. Compared to the normal control, cells with *Tmem53* knockdown expressed higher levels of osteoblast markers (*Bglap* and *Alpl*) in response to BMP2 stimulus (Fig. 5e). These results suggest that *Tmem53* deficiency enhances BMP signal-induced bone formation in calvaria cells.

To decide whether *TMEM53* regulates the SMAD-dependent BMP signaling pathway, we generated *TMEM53* knockout (KO) human cell lines by deleting the largest exon, which contains 78% of the coding sequence, in Hela cells (Supplementary Fig. 12). We used these cell lines in a BMP reporter assay^20^ and quantified SMAD-dependent BMP signaling activity. The assay showed that BMP signaling activity was upregulated in two independent *TMEM53* KO cell lines and was downregulated when *TMEM53* was overexpressed (Fig. 5f). Exogenous expression of *TMEM53* restored the BMP overactivity in *TMEM53* KO cell lines (Fig. 5f). These results demonstrate that TMEM53 plays an inhibitory role in the activation process of the SMAD-dependent BMP signaling pathway, suggesting the enhanced ossification in the calvaria of the *Tmem53* mutant mice and the individuals with *TMEM53* pathologic variants is caused by overactivation of the BMP signaling pathway.

### TMEM53 deficiency increased nuclear localization of phosphorylated SMAD1/5/9

To explore the mechanism that regulates overactivation of SMAD-dependent BMP signaling in the calvaria of the *Tmem53* mutant mice, we performed ICC for phosphorylated Smad1/5/9 in primary calvaria cells stimulated with BMP2. We found that phosphorylated Smad1/5/9 increased in the nucleus and correspondingly decreased in the cytoplasm (Fig. 5g, h), and those findings were supported by Western blot analysis of the cytoplasmic and nuclear protein extracts (Fig. 5i and Supplementary Fig. 13). In contrast, the whole-cell lysate did not show significant changes in the phosphorylation levels of Smad1/5/9 (Fig. 5j), and neither were Smad1/5/9/4 mRNA levels altered in calvaria RNA-seq data (Supplementary Fig. 14). These results suggest that overactivated BMP signaling in the calvaria of the *Tmem53* mutant mice can be attributed to the excessive trafficking of phosphorylated Smad1/5/9 into the nucleus rather than to their increased expression or phosphorylation levels. We performed ICC for phosphorylated SMAD1/5/9 in *TMEM53* KO Hela cells and observed the same trend as in the primary calvaria cells of the *Tmem53* mutant mice (Fig. 5k, l). Thus, the effect of the mutation in mice is equivalent to gene KO with respect to regulation of BMP signaling. Due to the phenotypic similarity between the *Tmem53* mutant mice and the individuals with *TMEM53* pathologic variants, it is reasonable to conclude that the previously unknown type of skeletal dysplasia is causally associated with loss-of-function of TMEM53 in regulating the cytoplasm–nucleus translocation of phosphorylated SMAD1/5/9 (Fig. 5m). This is further supported by the results of a rescue experiment in *TMEM53* KO cells, in which wild-type TMEM53, but not the truncated TMEM53 protein produced using the patient-derived pathogenic variant, succeeded in restoring the nuclear to cytoplasmic ratio of phosphorylated SMAD1/5/9 (Fig. 5k, l).

## Discussion

In this study, we discovered a previously unknown type of skeletal dysplasia with an autosomal recessive mode of inheritance. The observed skeletal changes have some similarities to three diseases belonging to the group of craniotubular dysplasias: craniometadiaphyseal dysplasia (CRMDD, MIM: %269300)^22^, craniometaphyseal dysplasia, Jackson type (CMD, MIM: #123000)^23^, and craniodiaphyseal dysplasia, Joseph-Halliday type (CDD, MIM: #122860)^24^. However, definite differences exist between our cases and patients with those SBDs. Although CRMDD also presents with sclerosis of the base of the skull, the calvaria show thinning rather than thickening. Multiple Wormian bones, dental hypoplasia, and frequent fractures are present in CRMDD, but no platyspondyly is found in CRMDD. CMD shows club-shaped metaphyseal widening of tubular bones, while our cases display quite mild metaphyseal dysplasias. Moreover, CMD does not show platyspondyly and under-constricted diaphysis, which are characteristic in our cases. CDD is by far more severe than our cases. CDD is lethal and shows severe craniofacial abnormalities, including distorted faces and prominent jaw. In addition, dental malocclusion and premature loss of teeth are observable in CDD. These obvious differences provide an operable guideline to separate the disorder reported here from the known skeletal dysplasias in clinical practice, supporting the consideration of this disorder as a previously unreported clinical entity that we suggest designating as craniotubular dysplasia, Ikegawa type.

So far, two skeletal disorders are known to be explicitly related to BMP-signaling: Myhre syndrome caused by *SMAD4* pathogenic variants (MIM: # 139210)^7^ and BMPR1B-related brachydactyly (MIM: # 609441, # 616849, # 112600)^8^, but both show no hyperostosis. The previously unknown disorder identified in this study shows a strong relationship with BMP signaling, thus probably being the only hyperostosis caused by overactivity of BMP signaling. As the other branch of TGF-β family signaling, overactivity of TGF-β signaling is considered to be the mechanism of Camurati–Engelmann disease (MIM: # 139210)^25^. The hallmark of Camurati–Engelmann disease is the cortical thickening of diaphyses of the long bones, which was not found in our cases.

In our study, multiple pieces of genetic and biological evidence from humans and mice indicate that the previously unknown type of SBD is caused by loss-of-function of TMEM53. TMEM53 deficiency leads to overactivation of the BMP signaling pathway, which plays key roles in craniofacial development^14, 15^. Overactivated BMP signaling during intramembranous ossification would cause hyperostosis with resultant calvarial thickening, which were observed in both human and mouse *TMEM5*3 mutants. Notably, besides the enhanced osteoblastogenesis in the mutant calvaria, we found an asymmetrical change in TRAP5 (tartrate-resistant acid phosphatase) staining (Fig. 4f), which suggests a defect in osteoclastogenesis, which probably also contributed to the dysregulated ossification. The defective osteoclastogenesis may be a change secondary to the defective osteoblastogenesis due to the close coupling of bone formation and resorption during calvarial remodeling^13^. Alternatively, the unequal distribution of TRAP staining is only due to the asymmetrical skull development, which is mirrored by the dysmorphic coronal sutures in the *Tmem53* mutant mice (Supplementary Fig. 15). Cranial sutures serve as growth centers composed of two osteogenic bone fronts on either side of the sutures. Maldevelopment of cranial sutures causes dysmorphogenesis of skulls in humans and mice^26^, and BMP signaling participates in the regulation of cranial suture morphogenesis^27^. Thus, the dysmorphic coronal suture can be linked to the overactivated BMP signaling and contribute to the craniofacial dysmorphism in human and mouse *TMEM53* mutants.

Besides cranial phenotypes, under-constriction or under-modeling in the meta-diaphyses of the mildly shortened tubular bones were another feature of the affected individuals with *TMEM53* pathogenic variants, and comparable phenotypes were evident in the *Tmem53* mutant mice as well (Fig. 3l, m and Supplementary Fig. 8). These findings suggest interrupted coordination of interstitial growth and appositional growth of tubular bones. Interstitial growth, also referred to as cartilaginous growth, elongates tubular bones by endochondral ossification in the growth plate. The specific expression pattern of *Tmem53* in the proliferative and pre-hypertrophic zones of the growth plate supports its functional involvement in the elongation of tubular bones (Supplementary Fig. 16a). In the *Tmem53* mutant mice, we observed a thickened growth plate in the femur (Supplementary Fig. 16b, c), and prior studies have shown that BMP signaling is important for growth plate regulation^6^. Together with our observations that *Tmem53* knockdown increased BMP2-induced chondrocyte markers in chondrogenic ATDC5 cells (Supplementary Fig. 16d–f), these results suggest *Tmem53* deficiency promotes chondrogenesis in the growth plate by overactivating BMP signaling. The enhanced chondrogenesis would disturb normal ossification in the growth plate and result in delayed growth in the length of tubular bones. In contrast to interstitial growth, appositional growth is a process of periosteal bone shaping, which determines the contour and diameter of tubular bones by coupling bone formative and resorptive activities beneath the periosteum. Unlike endochondral ossification in growth plates, periosteal bone shaping of tubular bones is similar to intramembranous ossification of the calvaria. The failure in the process manifests as “under-constriction” or “under-modeling” of meta-diaphyses in several known human bone dysplasias^3, 28, 29^. Additionally, *Tmem53* is highly expressed in the periosteal zone of tubular bones as well as in the calvaria (Supplementary Fig. 16a). These lines of evidence imply that the under-modeling of the meta-diaphyses of the tubular bones in human and mouse *Tmem53* mutants could be caused by a potential defect in periosteal bone shaping stemming from a molecular mechanism similar to that revealed for calvaria.

TMEM53 belongs to the family of NET proteins, which were identified based on their subcellular localization at the nuclear envelope^10^. As the boundary between the cell nucleus and cytoplasm, nuclear envelopes are formed by a specialized domain of the endoplasmic reticulum, where two closely juxtaposed lipid bilayers constitute a double-membrane sheet, the inner nuclear membrane (INM) and the outer nuclear membrane (ONM). Nuclear envelopes serve as a protective shell for the genome and a versatile communication interface between nucleus and cytoplasm. Pathogenic variants in NET proteins cause monogenic diseases, including muscular dystrophies, lipodystrophies, and neuropathy^30^. In addition, loss-of-function of an INM protein called the LEM domain containing 3 (LEMD3) results in a special SBD spectrum, osteopoikilosis (MIM: #166700)^31^. Our study provides the second case wherein deficiency of a NET protein induces SBD, thus reinforcing the functional link between NET proteins and the regulation of bone density. Besides SBD, *TMEM135*, which encodes another NET protein, was identified as a susceptibility gene for osteoporosis in several studies^32, 33^. These ever-growing lines of evidence suggest that the NET protein family could play hitherto unknown roles in bone metabolism as it relates to health and disease.

The study of TMEM53 function at the tissue and organism levels has not been reported previously. As far as we know, the only report about TMEM53 function is an in vitro research report indicating that TMEM53 affects cell cycle regulation in some cell lines^34^. However, the RNA-seq data of the calvaria of the *Tmem53* mutant mice did not show changes in proliferation markers (Fig. 4d). Also, the in vitro culture of the primary calvarial cells did not show any difference in the cell growth (Supplementary Fig. 9). The inconsistent findings imply that the effect of Tmem53 on cell cycles may depend on tissue and cell types as revealed by the previous report^34^. Instead, we found that TMEM53 deficiency promoted osteoblast differentiation by overactivation of BMP signaling. The BMP signaling pathway plays key roles in bone formation and development, and its intracellular regulation is realized by the modulation of the SMAD proteins. Our study reveals that TMEM53 prevents the nuclear accumulation of SMAD1/5/9 without affecting their levels of expression and phosphorylation. Nucleocytoplasmic transport of the SMAD proteins occurs through the nuclear pore complex (NPC)^35^. NPCs are assembled by multiple copies of ~30 different nucleoporins, which form a hydrophobic channel through the nuclear envelope. The trafficking process refers to multiple rounds of interaction among SMADs, transport receptors, and nucleoporins^35^. As an ONM protein^11^, TMEM53 might be engaged in these interactions to hamper SMAD1/5/9 translocation into the nucleus (Fig. 5m). The underlying mechanism by which TMEM53 functions as a gate-keeper of BMP-SMAD signaling at the nuclear membrane needs further study.

## Methods

### Patients

Individual A-V-1, a 19-year-old Indian male, was one of the two children of healthy and consanguineous parents (Fig. 1a). His birth and development were uneventful. Left orchiopexy has been performed at the age of 3 years. Short stature and mild coarsening of the face were recorded at age 5. He was first noted to have diminished vision at age 8 and completely lost vision in both eyes at age 10. He received decompression of optic nerves at age 10, which was not successful. He attained puberty at an appropriate age. He lost hearing in both ears at age 18. When he was age 19, his height was 150 cm (−3.6 SD), arm span 149 cm, lower segment 77 cm, occipital–frontal circumference (OFC) 55.5 cm, and weight 50 kg (−1.2 SD). He had prominent forehead, dolichocephaly, broad nasal root, anteverted nares, hypertelorism, epicanthic folds, thick vermillion of upper and lower lips, and prominent glabella (Fig. 1e, f). Ophthalmic evaluation revealed phthisis of the right eye and optic nerve atrophy of the left eye. Visual evoked potential test revealed no wave form in both eyes. The routine laboratory tests were normal. His skeletal survey showed thick calvaria, minor sclerosis of the skull base, broad ribs, mild platyspondyly, broad femoral neck, under-constriction of diaphyses of metacarpals and phalanges, and mild metaphyseal dysplasia of the knee joints (Fig. 1e, f, j, k, p–r and Supplementary Fig. 17a–d). Computed tomography (CT) of the orbits revealed diffuse thickening of calvaria with narrowing of the bony orbits and optic canals (Supplementary Fig. 17e). Right frontal orbitotomy was done for deterioration of vision. The subsequent imaging studies revealed progressive thickening of calvaria and constricting of all the foramina at the skull base. His brain was normal on imaging.

Individual A-V-2, a younger sister of A-V-1 (Fig. 1a), was born by a Cesarean section and weighed 2.45 kg at birth. She had a ventricular septal defect that closed spontaneously by 3 months of age. She had normal development and intelligence. She was apparently asymptomatic till age 10, when she was noticed to have short stature and shared characteristic facial features with her brother (Fig. 1g). Deterioration of her vision started at age 11. On the physical examination at age 12, her height was 138 cm (−2.7 SD), arm span 137.5 cm, lower segment 75 cm, OFC 52 cm, and weight 38 kg (−1.0 SD). Her hearing was normal. She had bilateral optic atrophy. Her routine laboratory examinations were unremarkable. Her skeletal survey showed quite similar abnormalities to her brother, including thickened calvaria, broad ribs, platyspondyly with relatively wide intervertebral disc, widened femoral neck, meta-diaphyseal dysplasias of short tubular bones of the hand, and mild metaphyseal dysplasia of the knee joints (Fig. 1l, m, s–u and Supplementary Fig. 18a–d). CT of the head revealed normal brain and narrow optic canals (Fig. 1i).

Individual B-III-18, was an 8-year-old girl, who sought medical consultation because of impaired vision within a few months of onset. Her birth, perinatal, and development history were unremarkable. The healthy parents were not consanguineous but were from the same village (Fig. 1b). Individual B-III-18 had a cousin (individual B-III-9) with the same disease (Fig. 1b). Her height was 107 cm (−3.6 SD), OFC 53 cm (normal), and weight 16 kg (−2.1 SD). She had normal intelligence. She had dolichocephaly, tall forehead, prominent eyes, hypertelorism, and epicanthic fold, thick vermillion of lips, broad nasal root, anteverted nares, and long philtrum (Fig. 1h). The hematological tests were normal. Skeletal survey showed thick calvaria, broad ribs, mild platyspondyly, and broad and short tubular bones of hands (Supplementary Fig. 19a–e). The long tubular bones of the upper limbs were normal (Supplementary Fig. 19f).

Individual C-II-1, a 15-year-old boy, sought medical consultation for diminished bilateral vision. His birth and perinatal history were uneventful. Parents were healthy and non-consanguineously married (Fig. 1c). His height was 148 cm (−3 SD), weight 42.7 kg (−3 SD), arm span 134 cm, and OFC 57 cm (+1.3 SD). He had macrocephaly, short hands, and small black colored nodules of 2–3 mm size on his face, and decreased vision. Eye evaluation revealed optic nerve compression, mydriasis, and cortical blindness. A skeletal survey showed thick calvaria, broad ribs, mild platyspondyly, broad and short tubular bones of hands, and mild metaphyseal dysplasia of knee joints (Fig. 1n–o, 1v–x and Supplementary Fig. 20a, b).

Individual D-III-4, a 17-year-old male, was referred to us due to progressive diminishment of bilateral vision during the past 7 months. His birth and perinatal history were uneventful. The healthy parents were cousins and he had an elder sister with similar disease (Fig. 1d). His height was 138 cm (−5 SD), weight 35 kg (−4 SD), and OFC 56 cm (+4 SD). He had developmental delay, dolichocephaly, hypertelorism, muddy conjunctiva, strabismus, bilateral gynecomastia, and syndactyly of the left third and fourth fingers. Skeletal survey showed thick calvaria, broad ribs, mild platyspondyly, broad and short tubular bones of hands, and mild metaphyseal dysplasia of knee joints (Supplementary Fig. 21a–f). Ophthalmic examination showed compressive optic neuropathy.

The authors affirm that human research participants have seen and read the material to be published and have provided informed consent for publication of the images in Fig. 1 and Supplementary Figs. 17–21.

### Whole-exome sequencing

The study was approved by the ethical committee of RIKEN, Kasturba Hospital, Manipal, and participating institutions (approval number: 17-16-40(3)). Genomic DNA were extracted by standard procedures from peripheral blood or saliva of the patients and/or their parents after informed consent. WES was performed as previously described^28, 29^. Briefly, genomic DNA (3 μg) was sheared by CovarisTM S2 system (Covaris) and processed using a SureSelectXT Human All Exon V5 kit (Agilent Technologies) or Nextera Rapid Capture Exome Kit (Illumina). We sequenced DNA captured by the kit using HiSeq 2000 (Illumina) or NextSeq500 Sequencer (Illumina). We performed the image analysis and base calling by HiSeq Control Software/Real Time Analysis and CASAVA1.6.0 or 1.8.2 (Illumina) and mapped the sequences to human genome hg19 by Novoalign (ver. 3.02.04) or BWA-MEM (ver. 0.7.15). We processed the aligned reads by Picard v.1.128 to remove PCR duplicate. Variants were called by Genome Analysis Toolkit (GATK) v2.7-4 based on GATK’s best practice Workflow v3 and annotated by ANNOVAR^36^.

### PCR and Sanger sequencing

Peripheral blood was obtained from the patient and their parents after informed consent. Genomic DNA and total RNA were extracted from the blood by standard methods. Genomic fragments containing the variants identified by WES were amplified by PCR and sequenced for both strands. Total RNAs (1 μg) were used to synthesize cDNA with a PrimeScript RT reagent Kit (Takara Bio). The electrophoretic bands of the PCR products were cut from the gel, purified, and sequenced. The PCR primer sets are shown in Supplementary Table 3. A 3730 DNA analyzer (Life Technologies) was used for the Sanger sequencing. Sequencher V.4.7 (Gene Codes) and Genetyx Ver.12 (Genetyx) were used for aligning sequencing chromatographs to reference sequences.

### Construction of expression plasmids

Clones for coding sequences of the three transcript variants of human *TMEM53* (GenBank: NM024587.4, NM001300746.1, NM001300747.2) were PCR-amplified from a cDNA library derived from human peripheral blood using KOD -Plus- (Toyobo). The PCR amplicons were cloned into the EcoRI and HindIII sites of the pTriEx4 expression vector (Novagen). The mutations were generated by inverse PCR-based site-directed mutagenesis kit (Toyobo). All PCR primers are shown in Supplementary Table 3.

### Immunofluorescence staining

MG63 or Hela cells in 8-well chamber slides (Nunc Lab-Tek, Thermo Scientific) were fixed in methanol:acetone (1:1) for 1 min and permeabilized with 0.3% Triton X-100–PBS for 10 min. The cells were blocked in 5% milk/10% FCS–0.3% bovine serum albumin/0.3% Triton X-100 in PBS for 30 min at room temperature. The staining was performed using anti-S-tag dylight 488 (1:500 dilution, ab117509, Abcam), Lamin A (1:1000 dilution, #86846, Cell Sgnaling Technology), anti-phospho-Smad1/5/9 antibody (1:100 dilution, #13820, Cell Signaling Technology), anti-rabbit IgG-fluor 546 (1:250 dilution, A11035, Invitrogen), anti-mouse IgG-fluor 546 (1:500 dilution, A11030, Invitrogen), and DAPI. The slides were mounted in Dako fluoresce mounting medium (Dako) and examined with a Nikon A1Rsi microscope (Nikon). The quantification of cytoplasm–nucleus translocation was performed using CellProfiler 3.0.0 with a customized analysis protocol of BBBC014v1 from the Broad Bioimage Benchmark Collection^37^.

### Generation of *Tmem53* mutant mice

All animal experimental protocols were approved by the Animal Experiments Committee of the RIKEN Center for Integrative Medical Sciences (approval number: 2019-009(5)) and conformed to institutional guidelines for the study of vertebrates. All animal experiments were carried out in accordance with the in-house guidelines for the care and use of laboratory animals of the RIKEN, Yokohama Institute, Japan. The mice were housed at a standard 12-h light/12-h dark cycle. The ambient temperatures is 21–25 °C with 40–60% humidity. For CRISPR/Cas9-mediated gene editing, guide RNAs (gRNAs) were designed using the GPP sgRNA Designer and CRISPR direct web sites. A DNA fragment containing T7 promoter followed by the gRNA sequence was PCR-amplified and cloned into the HindIII and XbaI sites of the pUC19 vector. The gRNAs were generated by T7 in vitro transcription (IVT) using MEGAshortscript Kit (Thermo Fisher Scientific). The Cas9 coding sequence was subcloned from pX330 (Addgene) into a pUC vector with T7 promoter. Using the linearized vector, Cas9 mRNA was synthesized by mMESSAGE mMACHINE T7 Ultra Kit (Thermo Fisher Scientific). Cas9 mRNA (20 ng/µl) and gRNAs (5 ng/µl) were dissolved in 10 mM Tris-HCl solution containing 0.1 mM EDTA (pH 8.0) and injected into the cytoplasm of fertilized eggs. The eggs were transferred to pseudo-pregnant female mice. Mutations in the offspring were identified by Sanger sequencing for tail tip genomic DNA. Mouse lines were established from founder mice carrying frame-shift mutations in targeted DNA sites. PCR primers for the plasmid construction and Sanger sequencing are listed in Supplementary Table 3.

### Skeletal preparation

After removing the skin and viscera, the mice were dehydrated with 100% ethanol for at least 3 days. The mice were stained with 0.03% Alcian blue 8 GX (Sigma) for 5 days and 0.002% Alizarin Red S (Sigma) in 1% KOH for 8 h. Then, the mice were cleared with 20% glycerol/1% KOH solution for several days and stored in 100% glycerol.

### ALP staining

For the whole-mount staining, mouse calvaria were dissected out and fixed in 4% PFA/PBS overnight. The calvaria were immersed in 1-Step NBP/BCIP solution (Thermo Scientific) for 5 h. The reaction was stopped by 4% PFA/PBS. For in vitro osteogenesis assay, primary calvaria cells were fixed in 4% PFA/PBS for 15 min and incubated with 1-Step NBP/BCIP solution for 20 min at room temperature. Images were captured using a MVX10 microscope equipped with a DP72 camera (Olympus).

### TRAP staining

TRAP staining solution was prepared by dissolving 0.01% naphthol AS-MX phosphate disodium (Sigma) and 0.06% Fast Red violet LB salt (Sigma) in TRAP buffer. The mouse calvaria were dissected out and fixed in 4% PFA/PBS overnight. Calvaria were immersed in the TRAP staining solution for 6 h at room temperature. Images were captured using a MVX10 microscope equipped with a DP72 camera (Olympus).

### Skeletal imaging by micro-CT and X-ray

The skeletal morphology of the adult mice was analyzed by micro-CT scans with Scan Xmate-L090 (Comscan) or Inspexio SMX-100CT (Shimadzu), and X-ray with TRS-1005 (SOFRON). Three-dimensional reconstruction and image processing were performed with ImageJ software or Avizo 6.3.

### In situ hybridization

A 500-bp *Tmem53* cDNA fragment was amplified by RT-PCR and cloned into the XhoI and BamHI sites of the pBluescript II SK(+) vector. The PCR primers are listed in Supplementary Table 3. Antisense and sense RNA probes for *Tmem53* were transcribed from the linearized plasmids with a digoxygenin (DIG) RNA labeling kit (Roche). For the whole-mount in situ hybridization, mouse embryos were fixed in 4% paraformaldehyde/PBS overnight and dehydrated with methanol. After rehydration, the embryos were treated with proteinase K (Invitrogen) and hybridized with DIG-labeled probes at 65 °C overnight. For the in situ hybridization analysis of frozen sections, the heads of mouse neonates were dissected out and fixed with 4% PFA/PBS containing 20% sucrose for 3 h. The specimens were then embedded in a cryo-embedding medium (Section-Lab) and frozen in *n*-hexane cooled with dry ice. Frozen sections (8-μm thick) were prepared using a cryostat microtome (Leica) and post-fixed with 4% PFA/PBS for 10 min at room temperature and carbethoxylated twice in 0.1% DEPC/PBS. Sections were hybridized with DIG-labeled probes at 55 °C overnight. After washing, the hybridized RNA probes were detected with ALP-conjugated anti-DIG antibody (Roche) and BM purple (Roche). Images were captured using a MVX10 microscope equipped with a DP72 camera (Olympus) and a CX41 microscope equipped with a DP20 camera (Olympus).

### RNA sequencing

After P3 mice were sacrificed by decapitation, the calvaria were immediately dissected out and preserved in RNAlater RNA Stabilization Reagent (Qiagen). The dissected calvaria were frozen in liquid nitrogen and crushed by a cryopress (Microtec). The homogenized tissue powders were dissolved in ISOGEN solution (Nippon Gene) followed by total RNA extraction using Qiagen RNeasy kit (Qiagen). RNA sample quality was assessed using an Agilent 2100 Bioanalyzer (Agilent Technologies). rRNA was removed by Ribo-ZeroTM Magnetic Gold Kit (Illumina). The cDNA was synthesized by NEBNext Ultra Directional RNA Library Prep Kit for Illumina R (New England biolabs). The libraries were fed into an Illumina NovaSeq 6000 sequencer according to the manufacturer’s instructions, with paired end reads and mean read lengths of 150 bp. Read quality was assessed using FastQC, and adapters were trimmed from the reads using FASTX-toolkit. RNA-seq yielded ~30 million reads per sample.

### Differential gene expression analysis of RNA-seq data

The reads from the RNA-seq were aligned to the mouse genome (NCBI build37.2) using Tophat. Aligned reads were quantified using Cufflinks. Differential gene expression analysis was performed with Cuffdiff. The volcano plot and the heatmaps were created using RStudio (ver. 1.0.153) and GraphPad Prism 8.0, respectively. GO term enrichment analysis was performed by DAVID 6.8.

### Primary cell isolation and in vitro osteogenesis assay

Primary calvaria cells were isolated by digesting the P3 mouse calvaria with 1 mg/ml collagenase A (Roche) for 20 min three times. The cells were expanded in α MEM (Sigma) supplemented with 10% FBS to 70–80% confluence prior to passaging. For in vitro osteogenesis assay, the passaged cells were cultured in osteogenic media containing osteoblast-inducer reagents (TAKARA) with or without 100 ng/ml BMP2 (Humankine) or 1.0 µM K02288 (CAY 16678). The differentiated cells were stained by 1% Alizarin Red S (Sigma) 95% ethanol and 1-Step NBP/BCIP solution (Thermo Scientific) for 20 min. Images were captured using a MVX10 microscope equipped with a DP72 camera (Olympus) and quantified using ImageJ. MC3T3-E1 and ATDC5 cells were cultured in α MEM (Sigma) supplemented with 10% FBS and DMEM/F12 (Sigma) supplemented with 5% FBS, respectively.

### Generation of *TMEM53* KO cell lines

Six gRNA sequences targeting genomic DNA within intron 2 or 3ʹ UTR of *TMEM53* were designed by E-CRISP. Complementary oligos containing the *TMEM53* guide sequences and *Bbs*I ligation adapters were annealed and ligated into the *Bbs*I-digested pX330 vector (Addgene). These oligo sequences are listed in Supplementary Table 3. Nine pairs of pX330 vector containing gRNA sequences were transfected into Hela cells by TransIT-LT1 (Mirus), respectively. Each pair of gRNA sequences targets intron 2 and 3ʹ UTR of *TMEM53*, respectively, thus leading to a deletion covering exon 3 of *TMEM53* (Supplementary Fig. 12a). The efficiencies of generating the deletions were detected by PCR for genomic DNA (Supplementary Fig. 12a). The pair of pX330 plasmids with the highest efficiency were selected and co-transfected with pEGFP-N1 (Clontech) vector, which was used as a fluorescent marker to sort transfected cells by FACSAria (BD Biosciences) (Supplementary Figs. 12b and 22). Monoclonal cell populations were obtained by limiting dilution of the sorted cells in half 96-well plate. The cell lines with deletions covering exon 3 of *TMEM53* were identified by PCR for genomic DNA (Supplementary Fig. 12c) and confirmed by Sanger sequencing (Supplementary Fig. 12d). RT-qPCR was performed to validate the KO effect (Supplementary Fig. 12e). PCR primers for the plasmid construction and Sanger sequencing are listed in Supplementary Table 3.

### Knockdown of *Tmem53*

Two Stealth siRNA-Tmem53 sequences (MSS229322 and MSS229323) were designed by BLOCK-iT™ RNAi Express and synthesized by Life Technologies Corporation (Invitrogen). The Stealth RNAi negative control medium GC duplex was used as a negative control. The siRNA were transfected using TransIT TKO (Mirus) according to the manufacturer’s protocol.

### Luciferase assay

The BMP reporter (pGL3 BRE Luciferase) plasmid was a gift from Martine Roussel and Peter ten Dijkewas (Addgene, #45126)^20^. The phRL-SV40 vector (Promega) was used as an internal control to normalize the variation in transfection efficiency. The wild-type Hela cell and two independent *TMEM53* KO cell lines were used for luciferase assay. Cells were seeded in 24-well plates at a density of 5 × 10^4^ and cultured at 37 °C under 5% CO_2_ in Dulbecco’s modified Eagle’s medium (DMEM) supplemented with 10% fetal bovine serum (FBS). After 24 h, 100 ng/ml BMP2 (Humankine) was added into the medium, and the cells were transfected using TransIT-LT1 (Mirus Bio) according the manufacturer’s instructions. The luciferase activities were measured using the PicaGene Dual Sea Pansy Luminescence kit (Toyo Ink).

### RT-qPCR

Total RNA from calvaria and cells was extracted using Qiagen RNeasy kit (Qiagen) and the SV Total RNA Isolation System (Promega), respectively, according to the manufacturer’s instructions. The cDNA was synthesized from total RNA using a PrimeScript RT reagent Kit (Takara Bio). We performed real-time qPCR with a StepOnePlus Real-Time PCR system (Applied Biosystems) and the QuantiTect SYBR Green PCR Kit (QIAGEN). The value of each mRNA expression was normalized to *Gapdh* in the same sample. The qPCR primers are listed in Supplementary Table 3.

### Western blot

Whole-cell lysates were harvested with 200 μl of RIPA Lysis and Extraction Buffer (Thermo Fisher Scientific). Cytoplasmic and nuclear extracts were prepared with NE-PER™ Nuclear and Cytoplasmic Extraction Reagents (Thermo Fisher Scientific). Western blots were performed by standard procedures using antibodies for Smad1 (1:1000 dilution, #9743, Cell Signaling Technology), phospho-Smad1/5/9 (1:1000 dilution, #13820, Cell Signaling Technology), β-actin-HRP (1:10,000 dilution, PM053-7, MBL, Japan), Lamin A (1:1000 dilution, ab226198, Abcam), β-tubulin (1:1000 dilution, H-235, Santa Cruz), rabbit IgG–peroxidase (1:20,000 dilution, A0545, Sigma-Aldrich), and S-protein HRP conjugate (1:5000 dilution, 69047, Novagen). The signals were detected by ECL Prime Western Blotting Detection Reagents (RPN2232, GE Healthcare). Quantification was performed using ImageJ software.

### Statistical analysis

Continuous variables were reported as mean ± SD and compared with a two-tailed Student’s *t*-test or a one-way ANOVA followed by a post-hoc Tukey’s multiple-comparison test as specified in the figure legends. *P* values < 0.05 were considered to be significant.

### Reporting summary

Further information on research design is available in the Nature Research Reporting Summary linked to this article.

## Supplementary information

Supplementary Information

Reporting Summary

## Data Availability

The human variant data have been deposited in NCBI ClinVar with the accession codes “SCV001446315” and “SCV001446316”. The variants were evaluated by using the databases: GeneBank (https://www.ncbi.nlm.nih.gov/genbank/), dbSNP (http://www.ncbi.nlm.nih.gov/projects/SNP/), ExAC (http://exac.broadinstitute.org/), gnomAD (https://gnomad.broadinstitute.org/), HGMD (https://portal.biobase-international.com/hgmd/pro/start.php), NNSPLICE (http://www.fruitfly.org/seq_tools/splice.html), ASSP (http://wangcomputing.com/assp/), HSF (http://www.umd.be/HSF/), and MaxEntScan (http://genes.mit.edu/burgelab/maxent/Xmaxentscan_scoreseq.html). Homozygosity mapping was performed by HomozygosityMapper (http://www.homozygositymapper.org/). The mouse RNA-seq data have been deposited in NCBI GEO with the accession code “GSE161193”. The patients’ genome data obtained by whole-exome sequencing are not publicly available because study participants did not give full consent for releasing data publicly. These data can be accessed under the condition that a joint research plan is made by the researchers and approved by the ethics committees. All the other data supporting the findings of this study are included within the article or the Supplementary information. Source data are provided with this paper.

## Source data

Source Data
