# Supplementary material for: Deficiency of TMEM53 causes a previously unknown sclerosing bone disorder by dysregulation of BMP-SMAD signaling
Source: Nat Commun. 2021 Apr 6;12:2046. doi: 10.1038/s41467-021-22340-8 (PMC8024261; doi:10.1038/s41467-021-22340-8)
Supplement: Supplementary file 1 — Supplementary Information [file 41467_2021_22340_MOESM1_ESM.pdf]

Supplementary Fig. 1

Chr	Position	dbSNP ID	REF	ALT	B-III-18	C-II-1	D-III-4
1	44679556	rs803369	A	G	G/G	A/A	G/G
1	45125970	N/A	GAA	–	–/–	–/–	–/–
1	45250007	rs59895800	C	G	G/G	G/G	G/G
1	45504720	rs7551698	G	C	C/C	C/C	C/C
1	45973928	rs2275276	G	A	A/A	A/A	A/A
1	46094027	rs1250	A	G	G/G	G/G	G/G
1	46095272	rs1135812	T	C	C/C	C/C	C/C
1	46159157	rs61735666	C	T	T/T	T/T	T/T
1	46195375	rs28375469	T	C	C/C	C/C	C/C
1	46476587	rs11211247	T	G	G/G	G/G	G/G
1	46655158	rs6659553	T	C	C/C	C/C	C/C
1	46660295	rs2292487	T	C	C/C	C/C	C/C
1	46743900	rs1048771	C	T	T/T	T/T	T/T
1	46746164	rs11542623	C	T	T/T	T/T	T/T
1	46774783	rs41292543	A	G	G/G	G/G	G/G
1	46827456	rs9865	A	G	G/G	G/G	G/G
1	47078637	rs4660947	T	C	C/C	C/C	C/C
1	47080665	rs11211328	C	T	T/T	T/T	T/T
1	47080679	rs6671527	G	C	C/C	C/C	C/C
1	47080741	rs12029680	C	T	T/T	T/T	T/T
1	47133811	rs11211337	T	C	C/C	C/C	C/C
1	47138819	rs614486	T	G	G/G	G/G	G/G
1	47139103	rs1025806	C	T	T/T	T/T	T/T
1	47395973	rs1126743	G	A	A/A	A/A	A/A
1	47571902	rs4926802	C	T	T/T	T/T	T/T
1	47571905	rs28463559	C	T	T/T	T/T	T/T
1	47607851	rs2056899	A	T	T/T	T/T	A/A

Common homozygous stretch of the three cases (2.4 Mb)

Supplementary Fig. 1: The region common in the homozygous stretches of the three patients. The genotypes are decided according to the whole exome sequencing data. Affected individual B-III-18, C-II-1 and D-III-4 have the common 2.4 Mb homozygous region (yellow) on chromosome 1 that contains *TMEM53*. The arrow shows the position of the mutation (c.62-5_62-3delTTC) identified in *TMEM53*.

Supplementary Fig. 2

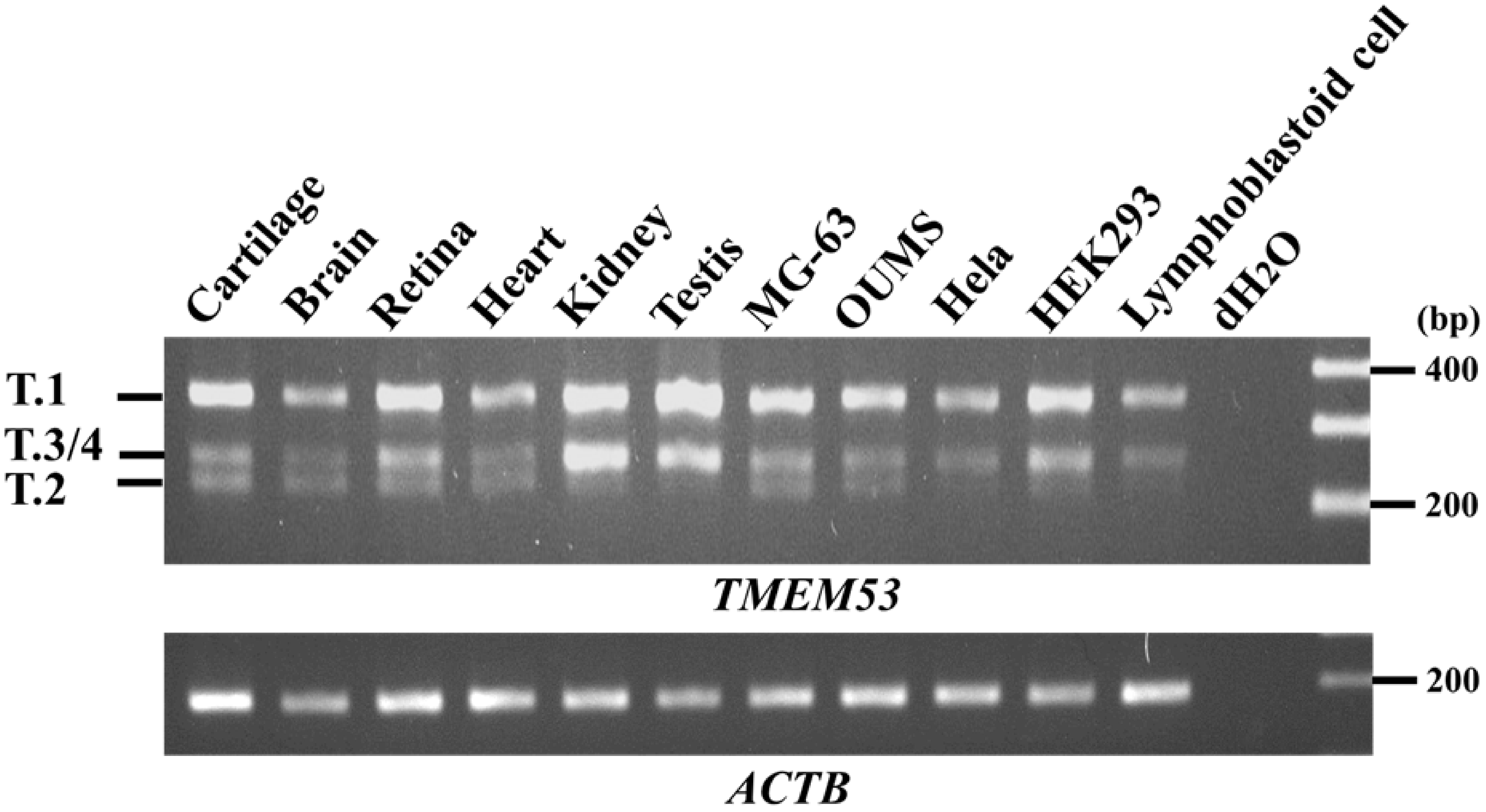

Supplementary Fig. 2: RT-PCR analysis for the *TMEM53* expression in multiple human tissue/cell types. *TMEM53* has four RefSeq transcripts. T.1, NM_024587.4; T.2, NM_001300746.1; T.3, NM_001300747.2; T.4, NM_001300748.2. T.3 has three more nucleotides than T.4. The transcript T.1 shows the highest expression in all tissue/cell types except for kidney, in which T.1 seems to be comparable with T.3/T.4. The PCR cycles are 30 for *TMEM53* and 24 for *ACTB*. n=2 independent experiments.

Supplementary Fig. 3

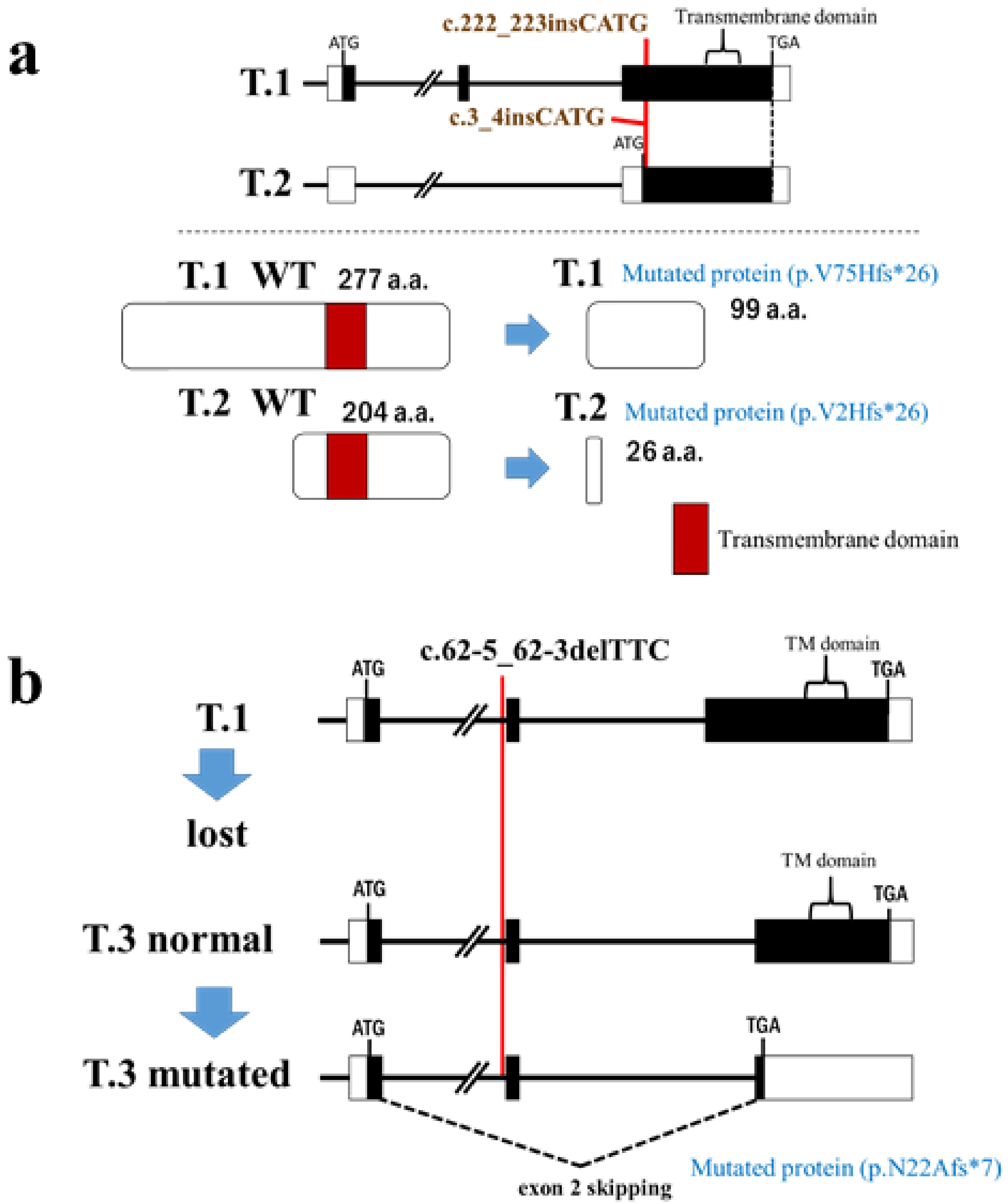

Supplementary Fig. 3: The effects of the two *TMEM53* variants at protein levels.

TMEM53 has four RefSeq transcripts, T.1 (NM_024587.4), T.2 (NM_001300746.1), T.3 (NM_001300747.2), and T.4 (NM_001300748.2). **a**, The variant c.222_223insCATG is located in the coding sequences of T.1 and T.2, thus causing a frame-shift in exon 3. Because the exon 3 of *TMEM53* is the last exon, nonsense-mediated mRNA decay would not occur. Instead, the mutated T.1 and T.2 produce two truncated proteins, p.V75Hfs*26 and p.V2Hfs*26, respectively, which lack the transmembrane domain. **b**, The variant c.62-5_c.62-3delTTC neighbors the intron 1-exon 2 junction and causes abnormal splicing, which leads to T.1 loss and exon 2 skipping in T.3. As a result, a premature stop codon was generated in exon 3, resulting in a truncated protein, p.N22Afs*7, which also lacks the transmembrane domain.

Supplementary Fig. 4

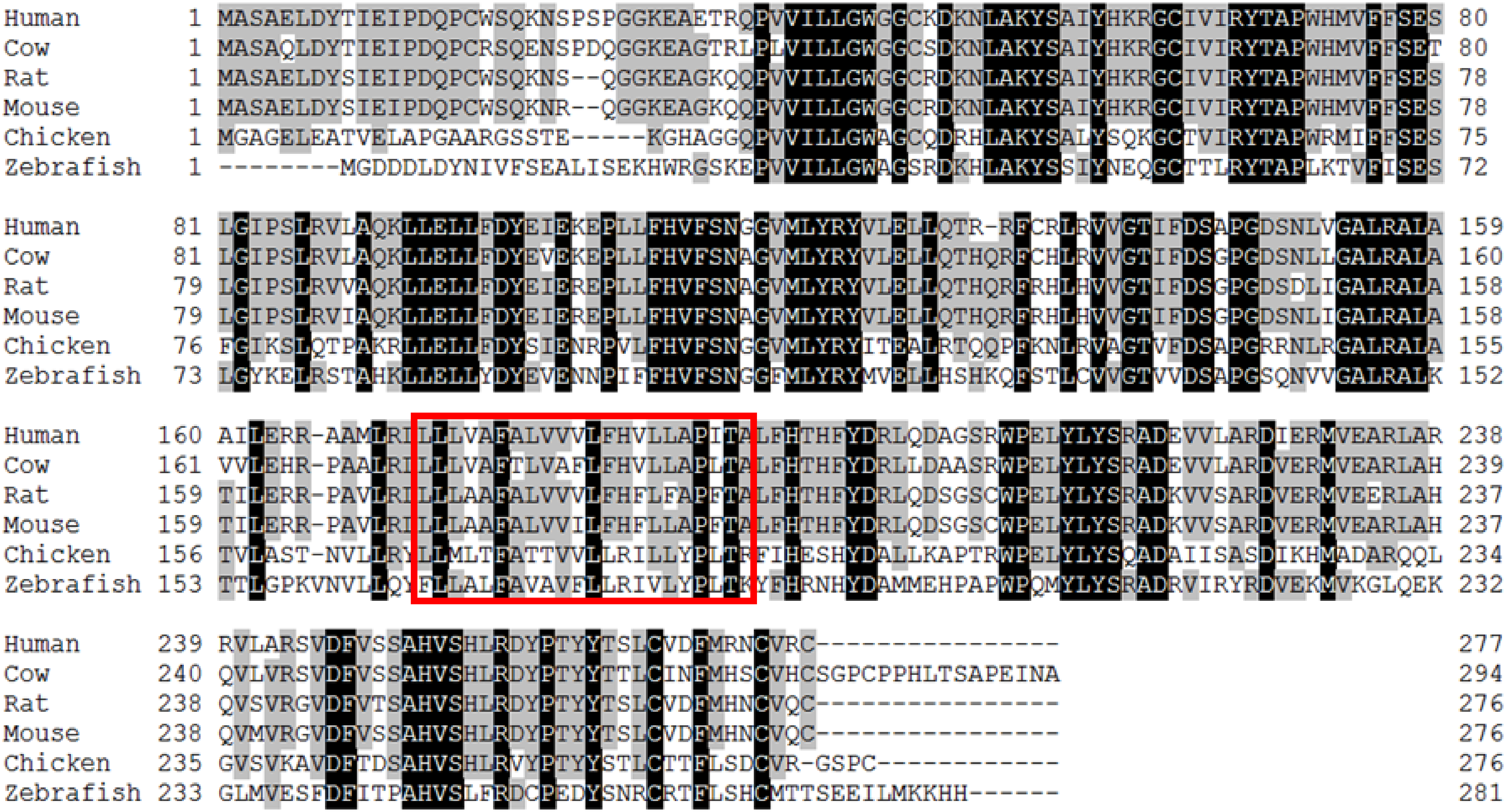

Supplementary Fig. 4: Multiple alignment of the amino acid sequences of TMEM53 from different species. TMEM53 is highly conserved among diverse species and has an 86.3% identity between human and mouse. The transmembrane domain is boxed in red. The sequences alignment and the identity calculation were performed by ClustalW2.

Supplementary Fig. 5

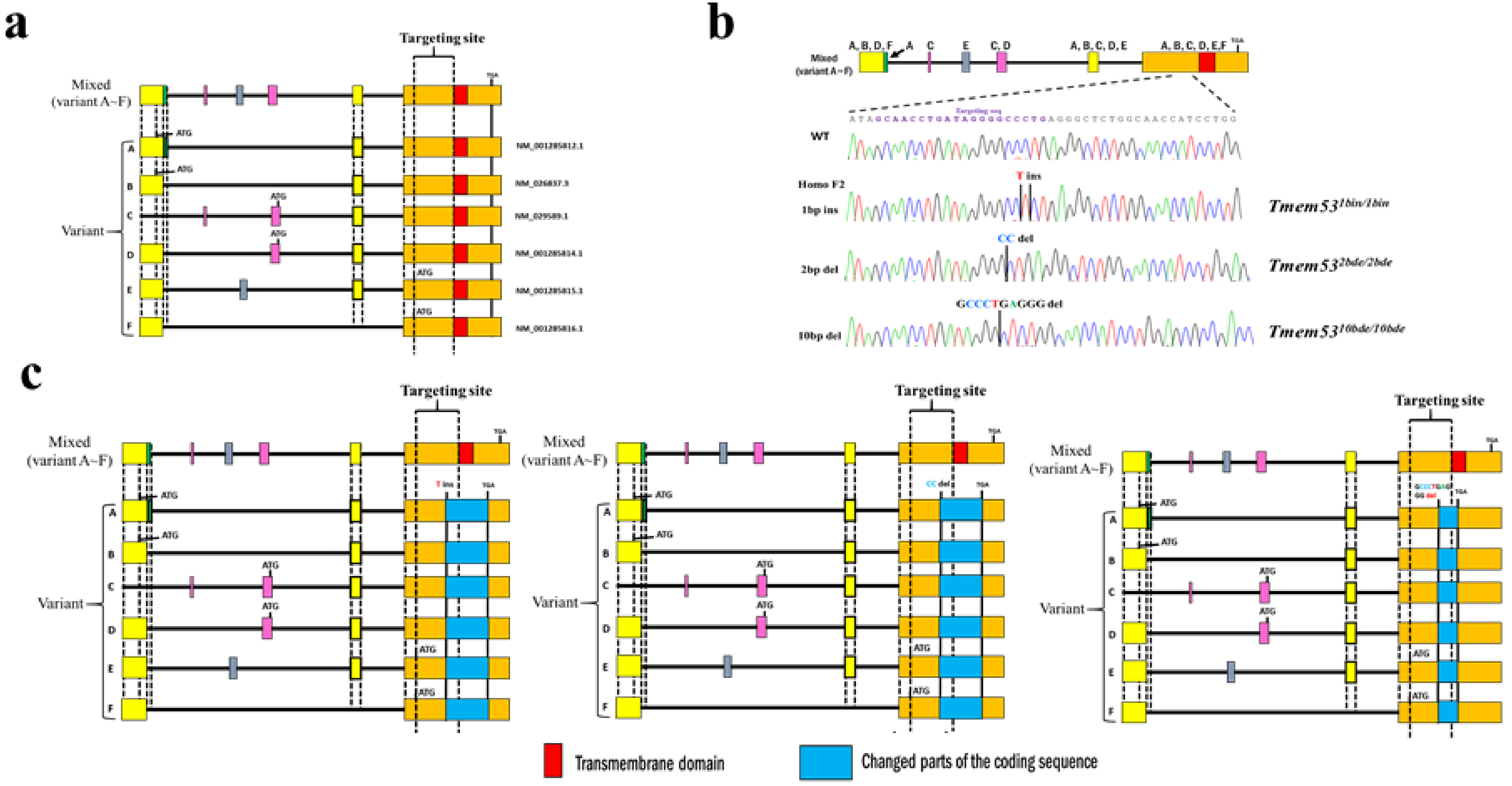

Supplementary Fig. 5: Generation of *Tmem53* mutant mice by CRISPR/Cas9-mediated gene editing.

a, A targeting site shared by all six RefSeq transcripts of *Tmem53* was selected for the gene editing. The colored boxes indicate the exons. **b**, DNA chromatographs for sequences of a wildtype (WT) and three established mutant lines in founder 2 (F2). The three lines (1 bp insertion, 2 bp deletion and 10 bp deletion) present different frame-shift mutations. **c**. The three mutations produce truncated proteins without the transmembrane domain in all transcripts.

Supplementary Fig. 6

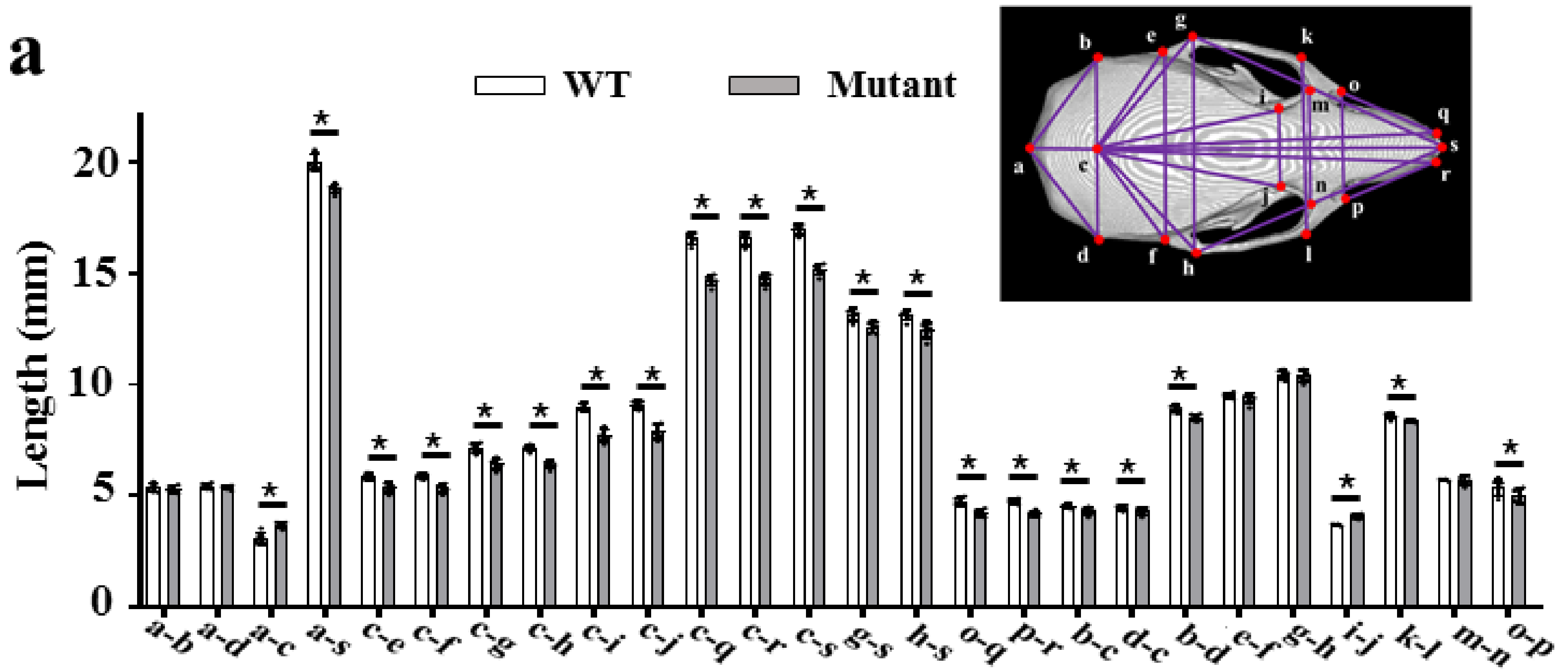

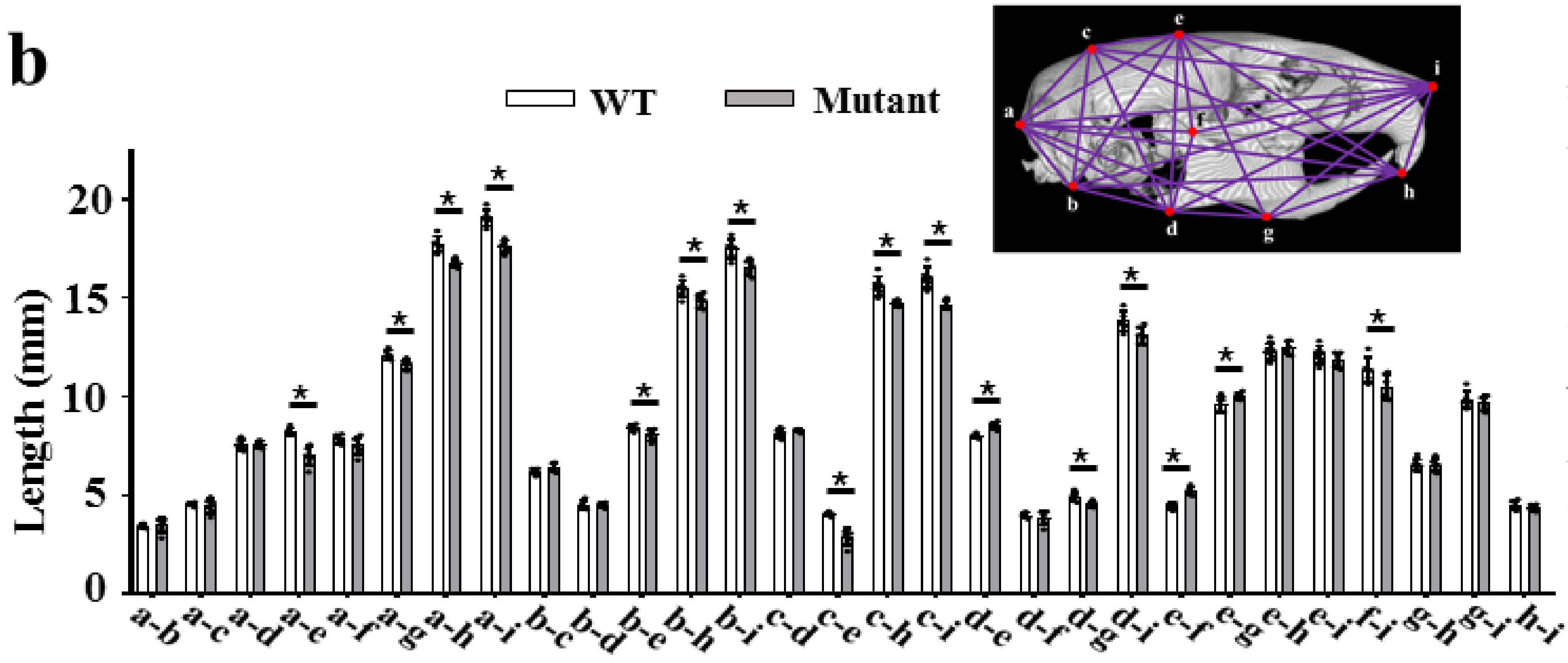

Supplementary Fig. 6: Measurement of cranial dimensions of the *Tmem53* mutant mice.

a, The distances between cranial landmarks (a-s) at the dorsal view. **b,** The distances between cranial landmarks (a-i) at the lateral view. Data indicate mean ± SD (n=6 wild type (WT) mice versus *Tmem53* mutant mice). Statistical significance was assessed using two-sided t test.*FDR-corrected *P* value < 0.05. The exact *P* values are included in the Source Data file.

Supplementary Fig. 7

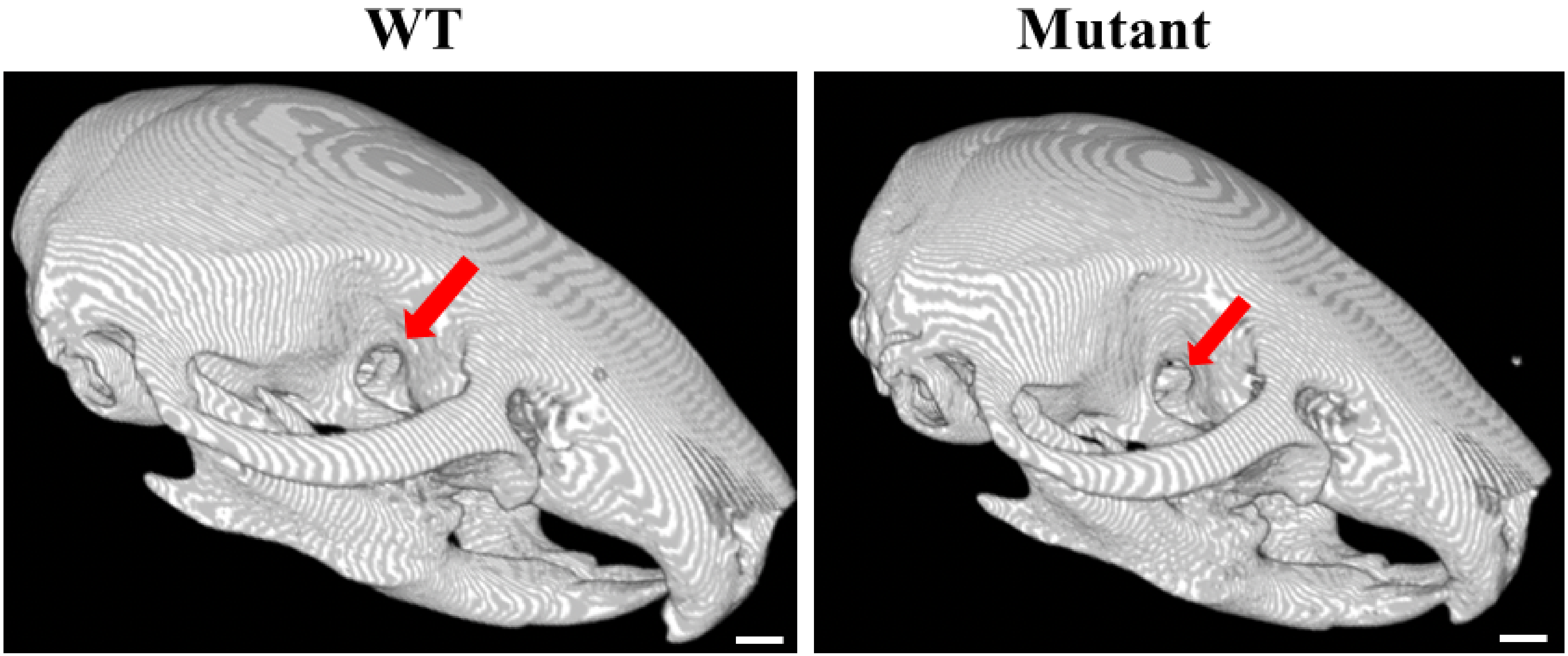

Supplementary Fig. 7: Dysmorphic bony fissure disturbing the optic foramen.

The bony fissure (red arrow) through which the optic nerve goes narrowed in the *Tmem53* mutant mice (Mutant), compared to the wild type mice (WT). n=3 mice. Scale bar, 1 mm.

Supplementary Fig. 8

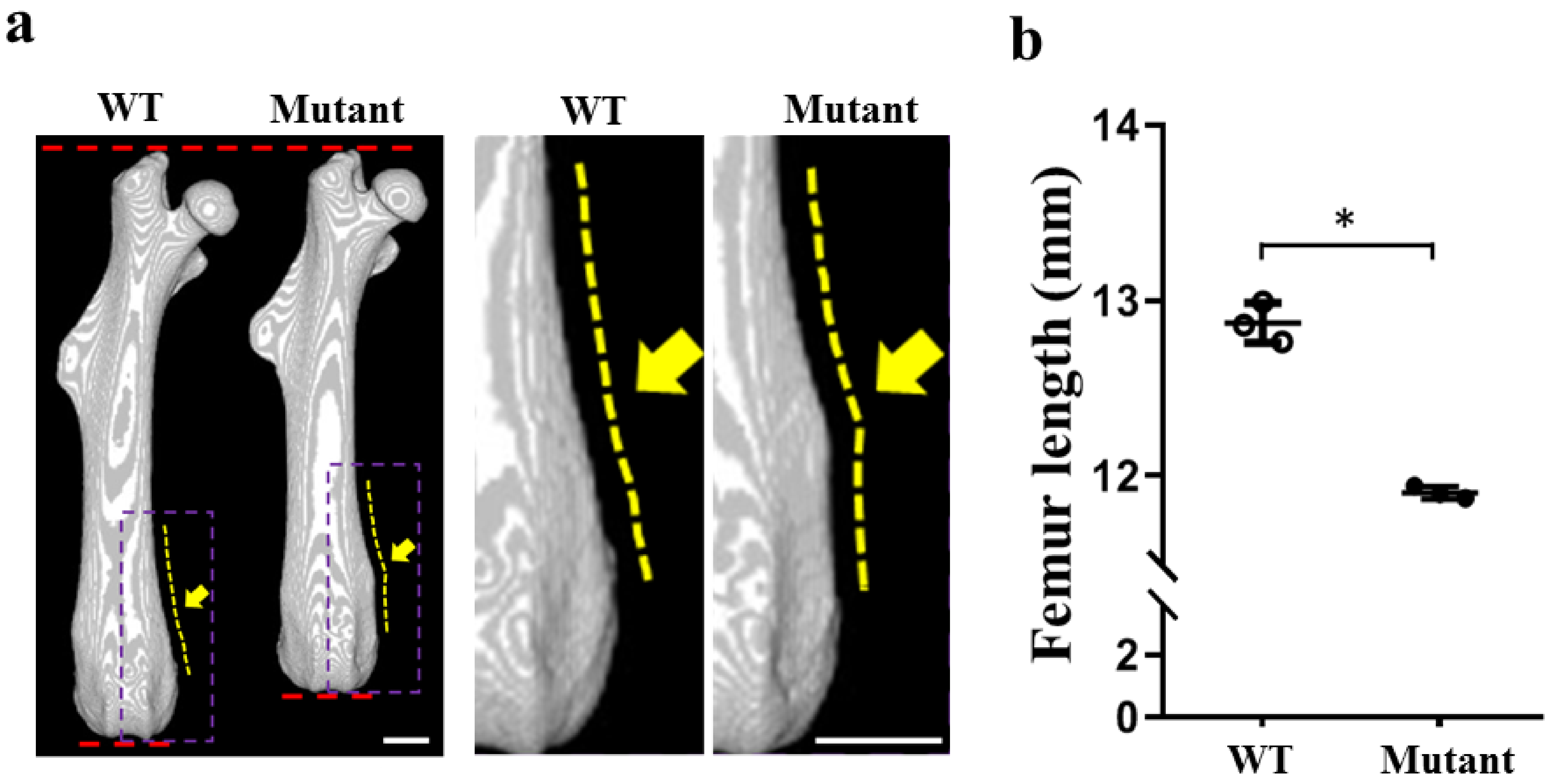

Supplementary Fig. 8: Shortening of the long tubular bone in adult *Tmem53* mutant mice.

a, Representative 3D images reconstructed from micro-CT stacks of P113 mouse femurs. The right panel is the enlarged view of the left panel. The yellow dotted lines drawing the contour of the meta-diaphyseal regions display under-constriction (arrow) in the femur of *Tmem53* mutant mouse. WT, wild type. Scale bar, 1 mm. **b**, Measurement of femur length. Data are mean ± SD (n=3 male mice). Statistical significance was assessed using two-sided t test, *$P = 0.0002$.

Supplementary Fig. 9

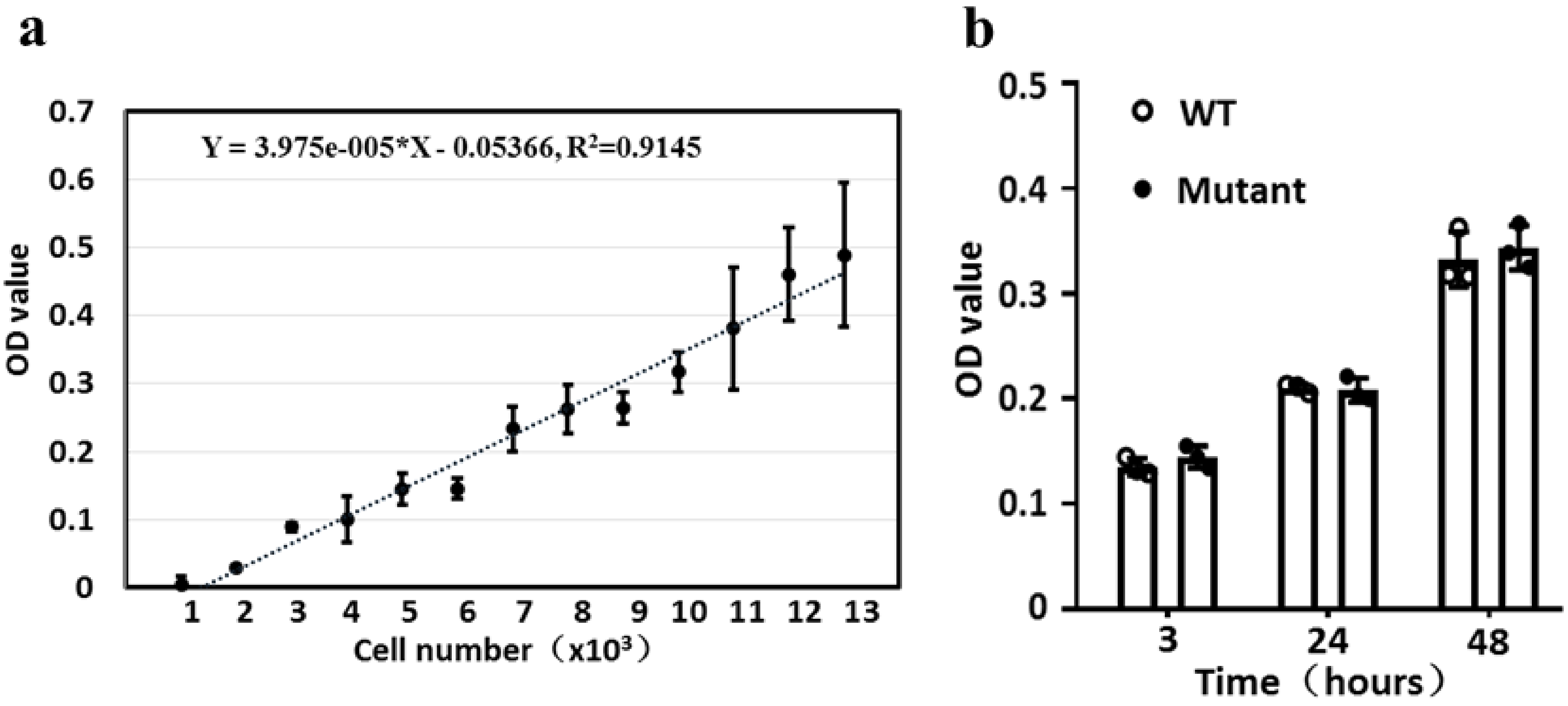

Supplementary Fig. 9: Growth of the primary calvaria cells by an MTT assay.

a, A linear correlation between the cell number of the primary cells extracted from mouse calvaria and the optical density (OD) measured at 540 nm. n=3 biologically independent samples. Data indicate mean ± SD. A linear regression line was obtained between the cell numbers and the OD value: OD value = 3.975e-005 × [cell numbers] – 0.05366, with the slope coefficient different from 0 ($P < 0.0001$). This model accounted for 91.5% of the OD value variance. **b**, Comparison of the numbers of the calvaria cells between wildtype (WT) and *Tmem53* mutant mice. n=3 biologically independent samples. Data indicate mean ± SD. Statistical significance was assessed using two-sided t test. $P = 0.2903, 0.7896, 0.6018$ for 3, 24, 48 hours culture, respectively.

Supplementary Fig. 10

	DEGs	Genome
Target genes of Smad1/5/4	429 (19.23%)	2953 (17.37%)
Non-target genes of Smad1/5/4	1802 (80.77%)	14047 (82.63%)

Fisher's exact test   Two-sided *P* value = 0.0309

Supplementary Fig. 10: Enrichment analysis of target genes of Smad1/5/4 in the *Tmem53* mutant calvarias. The list of the differentially expressed genes (DEGs) was generated from the RNA-seq dataset of calvarias of *Tmem53* mutants and their heterozygous littermates (n=3 animals for each group). It included 2,231 significantly DEGs (FDR-corrected *P* value < 0.05). The target gene list of Smad1/5/4 was obtained from the published data (Fei, *et al*. Genome Research 2010). Statistical significance was assessed using Fisher's exact test.

Supplementary Fig. 11

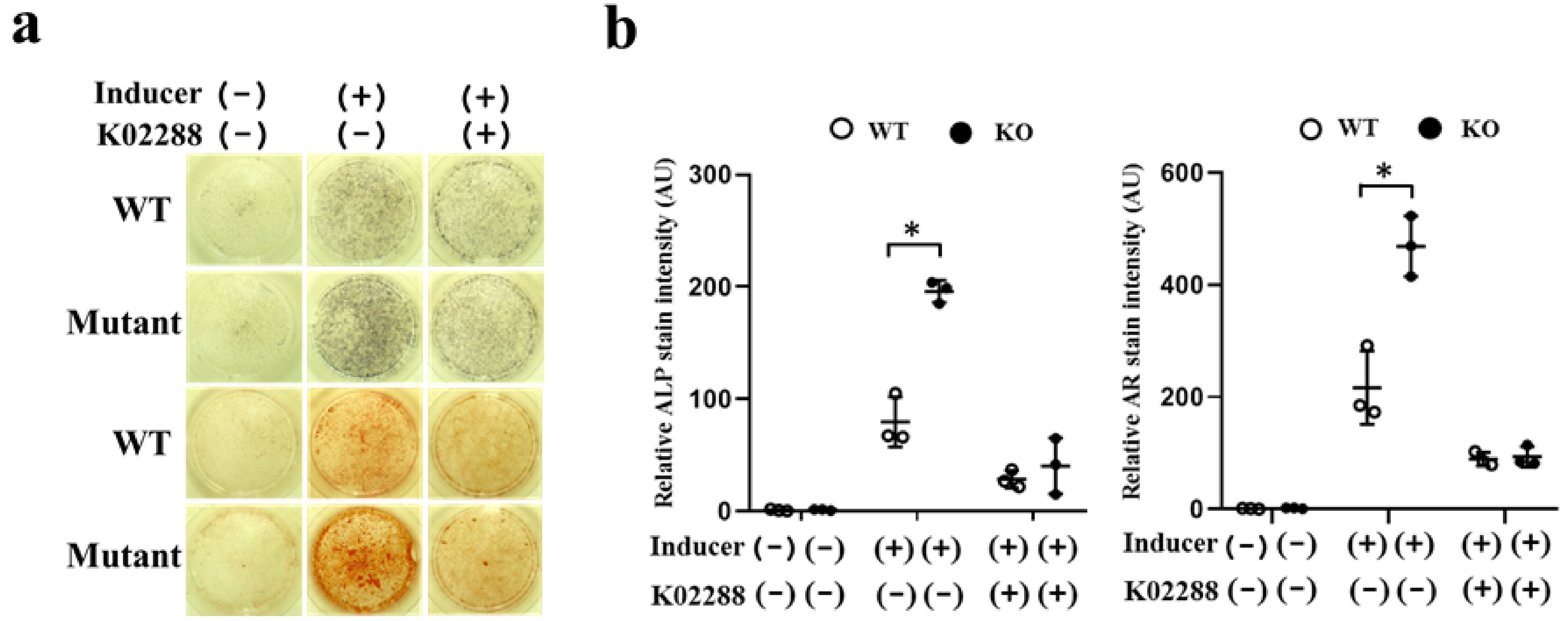

Supplementary Fig. 11: Inhibition of BMP signaling ablates the enhanced osteoblastic activity and bone formation in the *TMEM53* mutant calvarial cells. a, Alkaline phosphatase (ALP) and alizarin red (AR) staining for osteo-induced primary calvarial cells from the wild type (WT) and *Tmem53* mutant (KO) mice with or without addition of K02288, a selective and potent inhibitor of the BMP type I receptor kinases. Inducer: ascorbic acid, hydrocortisone and β-glycerophosphate. **b**, Quantification of ALP and AR staining. The integrated optical density of WT without osteo-induction was set at 1. All data were presented as means ± SD (n=3 biologically independent samples). Statistical significance was assessed using two sided t test. *P* value is adjusted by false discovery rate (FDR). *P* = 0.408179, 0.002414 * and 0.408179 for ALP staining. P = 0.266135, 0.014170* and 0.525815 for AR staining. AU, arbitrary unit.

Supplementary Fig. 12

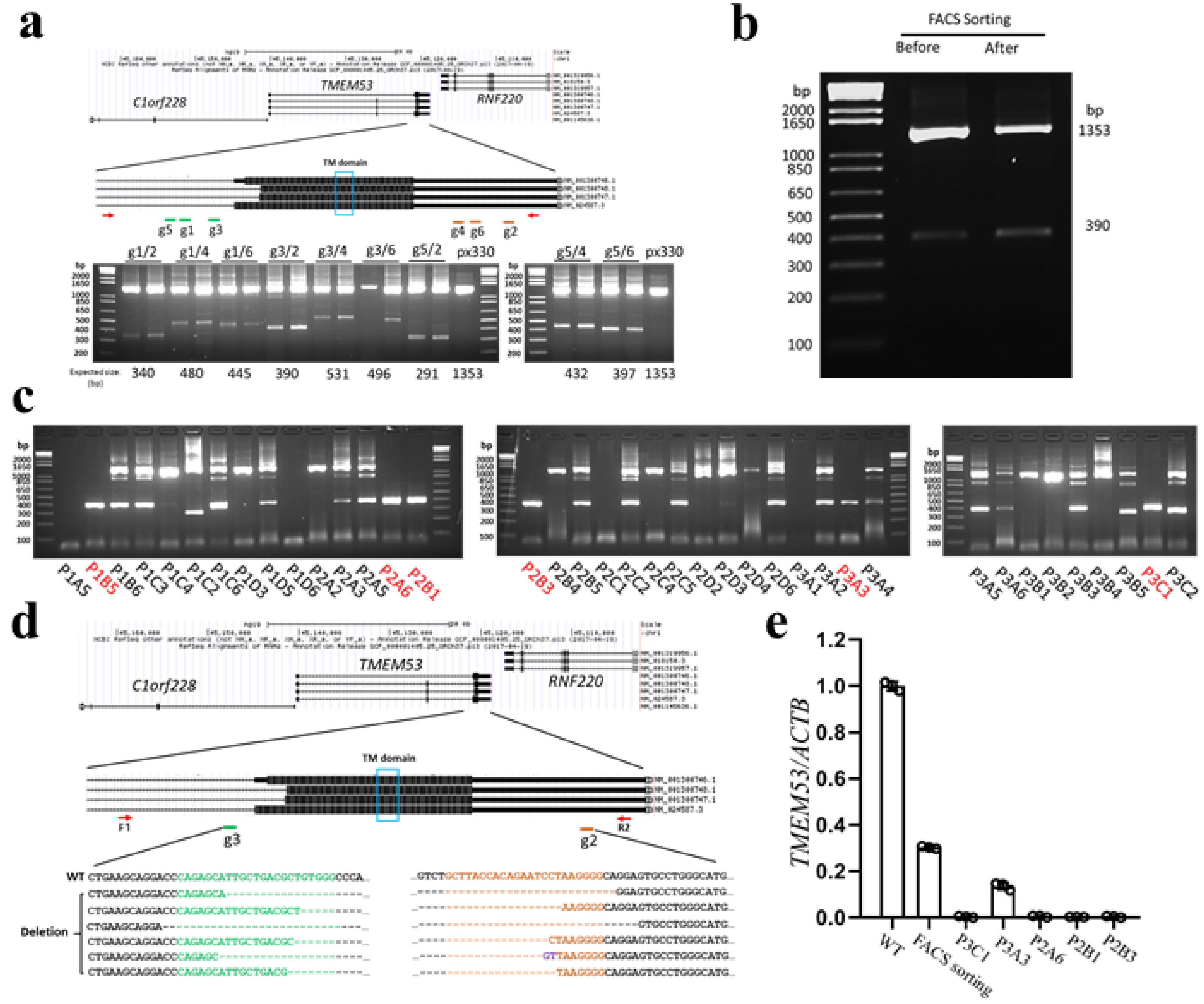

Supplementary Fig. 12: Generation of *TMEM53* knock-out Hela cell lines by CRISPR/Cas9-mediated gene editing.

a, Upper panel: Six guide RNAs (gRNA, g1-6) were designed to target genomic DNA within intron 2 or 3' UTR of *TMEM53*. Nine pairs of gRNA flanking exon 3 were introduced into Hela cells with Cas9. Lower panel: The small bands in the PCR using the primer set (red arrow) reflect the efficiencies of generating a large deletion including exon 3 by the nine pairs of gRNAs. **b,** The proportion of the cells with a deletion generated by g2 and g3 was increased after FACS sorting. **c**, Monoclonal screening for *TMEM53* knock-out lines. The clones without the PCR band corresponding to wild type (marked in red) were selected as candidate monoclonal lines. The screening for the most efficient gRNA in **a** and the candidate monoclonal cell lines in **b** and **c** was performed once. **d,** Sanger sequencing confirming the genomic deletion in these candidate lines. **e**, RT−qPCR-based validation of the knock-out effect in these lines. n=3 biologically independent samples. Data indicate mean ± SD.

Supplementary Fig. 13

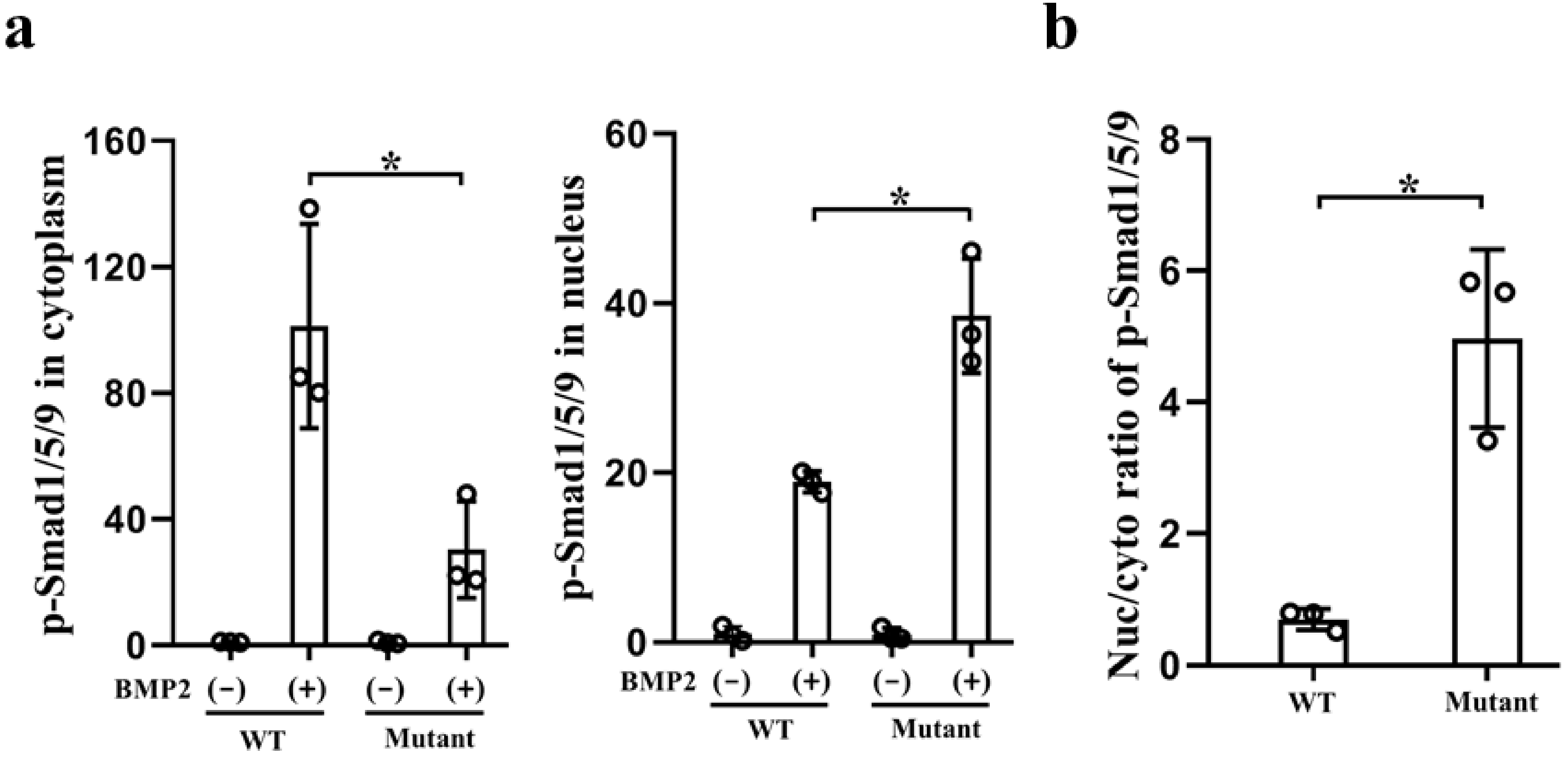

Supplementary Fig. 13: Quantification of Western blot of cytoplasmic and nuclear fractions of the wild type (WT) and mutant primary calvaria cells. a, The relative amount of p-Smad1/5/9 in cytoplasm and nucleus. Data are expressed as arbitrary units indicating mean ± SD of three independent Western blot experiments. Statistical significance was tested with two-sided t test, *$P = 0.0266$ and 0.0078. **b**, Nuclear to cytoplasmic (nuc/cyto) ratio of p-Smad1/5/9 in BMP2-stimulated WT and mutant primary calvaria cells. Data indicate mean ± SD of three independent Western blot experiments. Statistical significance was tested with two-sided t test. *$P = 0.0055$.

Supplementary Fig. 14

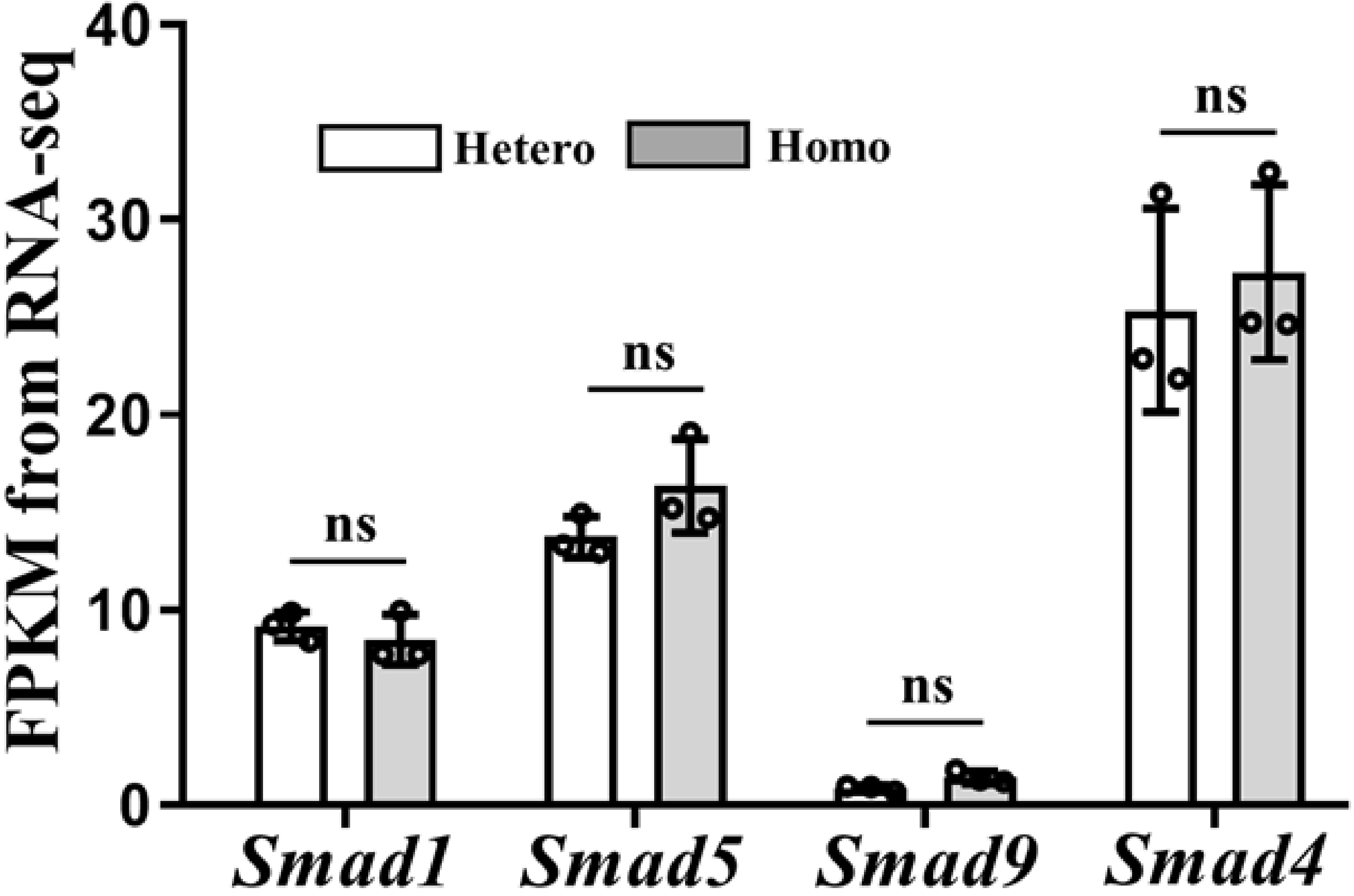

Supplementary Fig. 14: Smad1/5/9/4 expression levels in mouse calvarias. The mRNA levels in calvarias of *Tmem53* mutants (Homo) and their heterozygous littermates (Hetero) were determined by RNA-seq in FPKM units (n=3 animals for each group). Data are mean ± SD (n=3 mice). FPKM, fragments per kilobase of exon per million reads mapped. Statistical significance was assessed using a two-tailed Student's. ns, no significance.

Supplementary Fig. 15

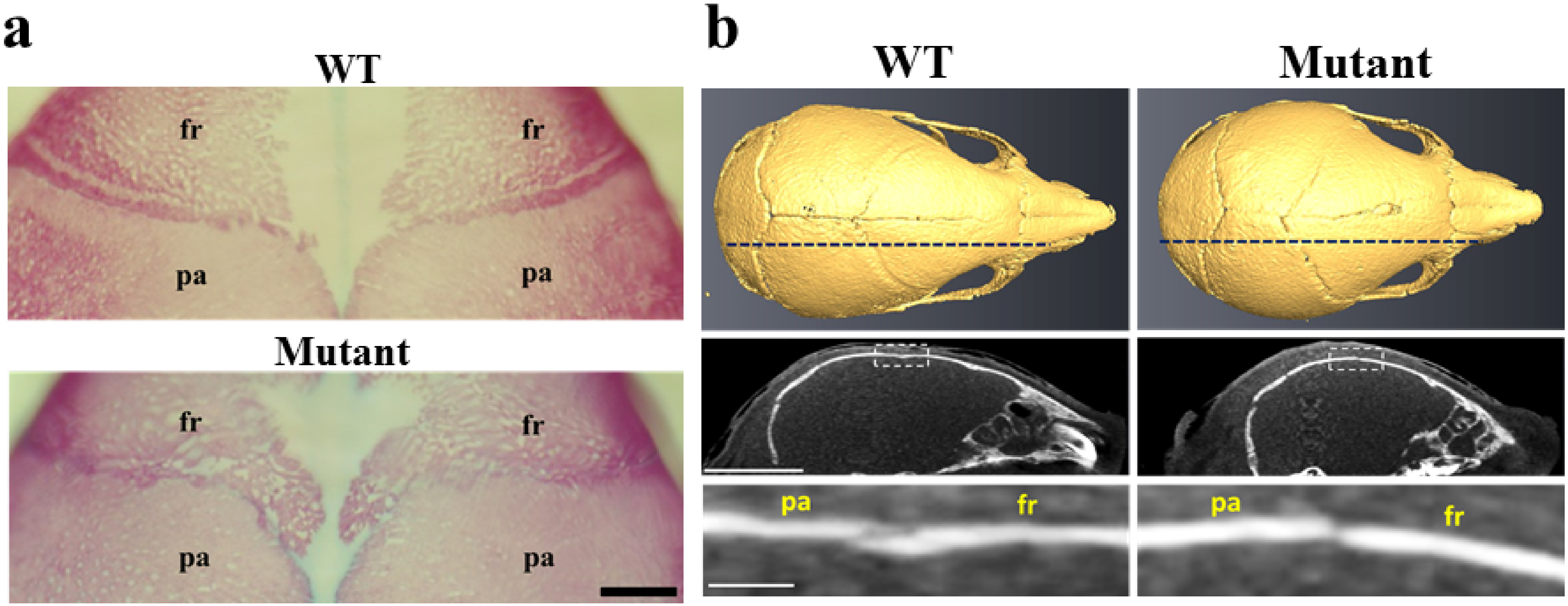

Supplementary Fig. 15: Dysmorphic coronal sutures of the *Tmem53* mutant mice. a, Dorsal view of the dissected calvarias stained by alizarin red at P2. The coronal sutures of the *Tmem53* mutant mouse (Mutant) show a unstraight shape compared with that of the wild type (WT) mouse. fr, frontal bone; pa, parietal bone. n = 3 mice. Scale bar, 1 mm. **b,** micro-CT of mouse skulls at P14. The upper panels are the 3D images reconstructed from the micro-CT stacks. The coronal sutures are asymmetrical in the mutant mouse. The middle panels are the sagittal micro-CT images on the planes indicated by the dotted line in the upper panels. n = 2 mice. Scale bar, 5 mm. The areas highlighted by the dotted box in the middle panels are magnified in the lower panels. Scale bar, 500 µm. At the coronal sutures, the frontal bone (fr) and the parietal bone (pa) show a head-to-head arrangement in Mutant compared with the overlapped bony plates in WT.

Supplementary Fig. 16

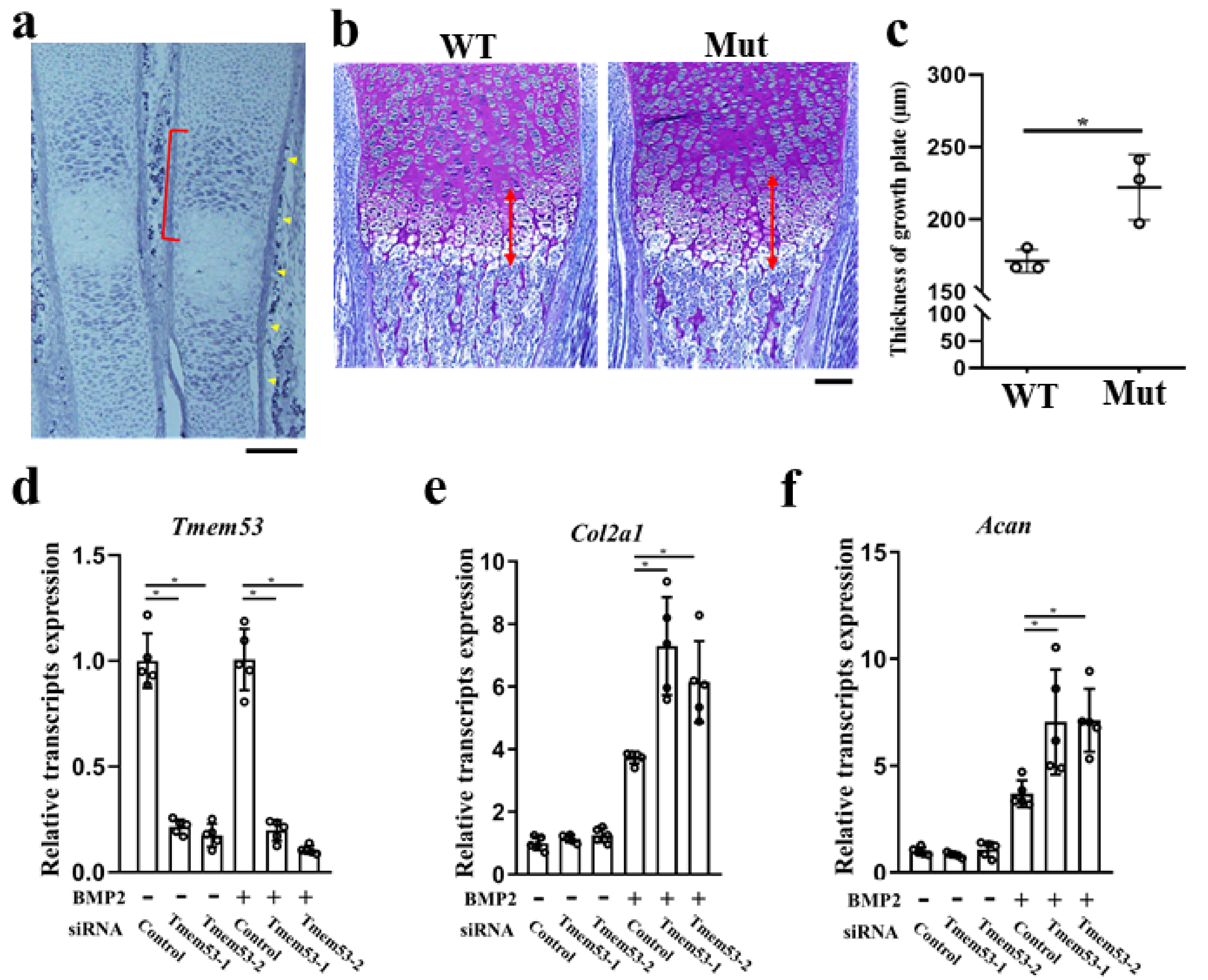

Supplementary Fig. 16: *Tmem53* deficiency affects chondrocyte development in growth plate. a, *In situ* hybridization for *Tmem53* in a E18.5 mouse forepaw shows specific expression of *Tmem53* in the proliferative and pre-hypertrophic zones of the growth plate (red bracket) and the periosteal zone of the tubular bone (yellow arrow head). Scale bar, 100 µm. n = 2 independent experiments. **b**, Toluidine blue staining for the sagittal section of P0 mouse distal femur. Scale bar, 100 µm. n = 3 mice. **c**, Measurement of the growth plate thickness in panel b. n = 3 mice. Statistical significance was assessed using two sided t test, $*P < 0.0215$. **d**-**f**, Effects of *Tmem53* knockdown by siRNA on the mRNA expression of BMP2-induced chondrocyte maker genes in ATDC5 cells (n=5 biologically independent samples). Compared to control siRNA, two independent sets of siRNA targeting *Tmem53* promoted *Col2a1* and *Acan* expression induced by BMP2. Statistical significance was tested with one-way ANOVA, *Tmem53*: $*P = 4.0 \times 10^{-12}$ and 1.348×10^{-12} and for control vs. knock-down without BMP; $*P = 2.1 \times 10^{-12}$ and 2.2×10^{-13} for control vs. knock-down with BMP. *Col2a1*: $*P = 9.6 \times 10^{-6}$ and 1.7×10^{-3}. *Acan*: $*P = 2.4 \times 10^{-3}$ and 1.8×10^{-3}. The data in **c**-**f** indicate mean ± SD.

Supplementary Fig. 17

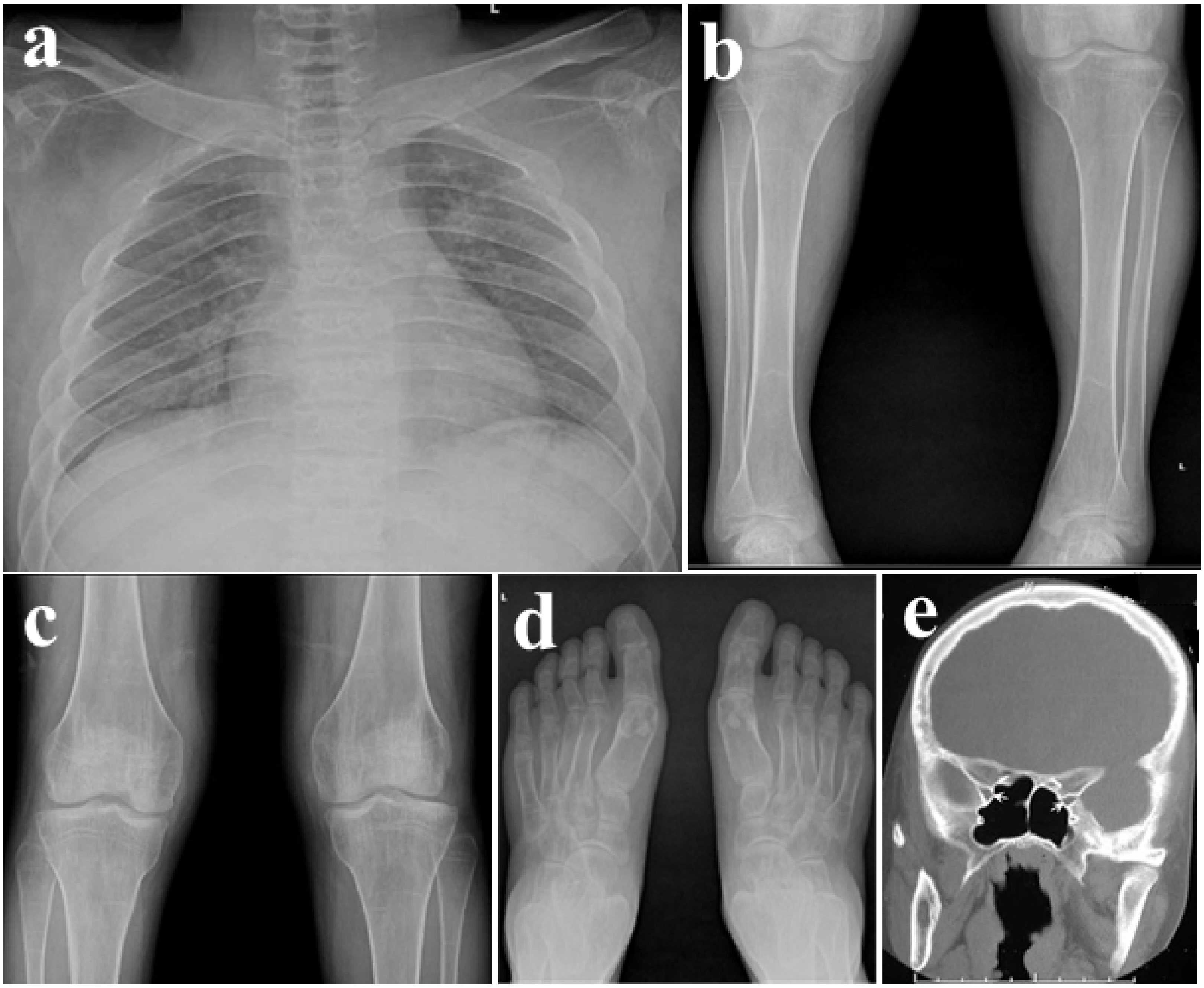

Supplementary Fig. 17: Radiographs of Individual A-V-1.
a, Broad ribs. **b-c,** Mild metaphyseal dysplasia of knee and ankle joints. **d,** Broad and short tubular bones of feet. **e,** CT image shows narrowing of optic canal.

Supplementary Fig. 18

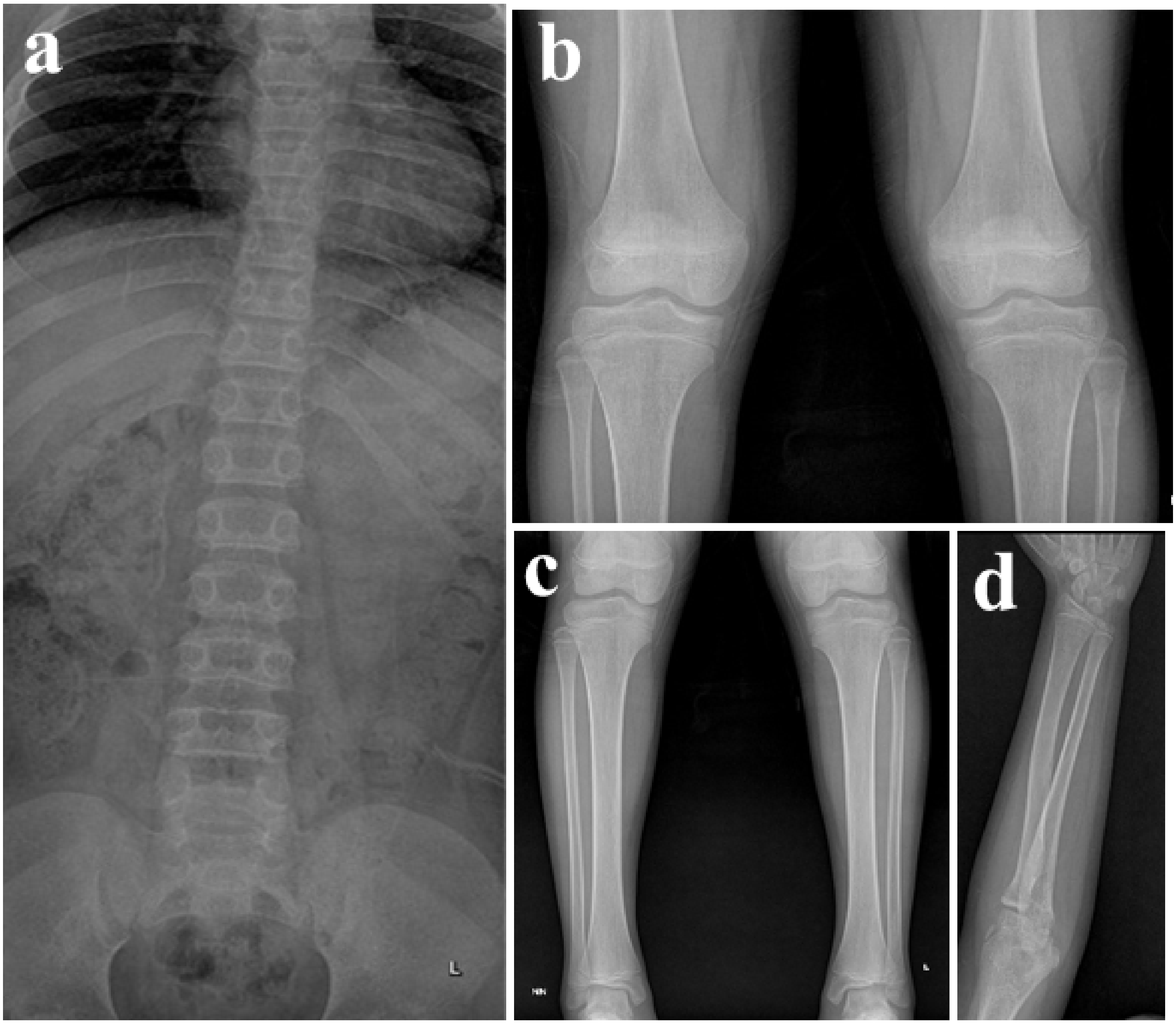

Supplementary Fig. 18: Radiographs of Individual A-V-2.
a, Broad ribs and mild platyspondyly. **b-c,** Mild metaphyseal dysplasia of the knee and ankle joints.
d, Mild metaphyseal dysplasias of the tubular bones of upper limbs.

Supplementary Fig. 19

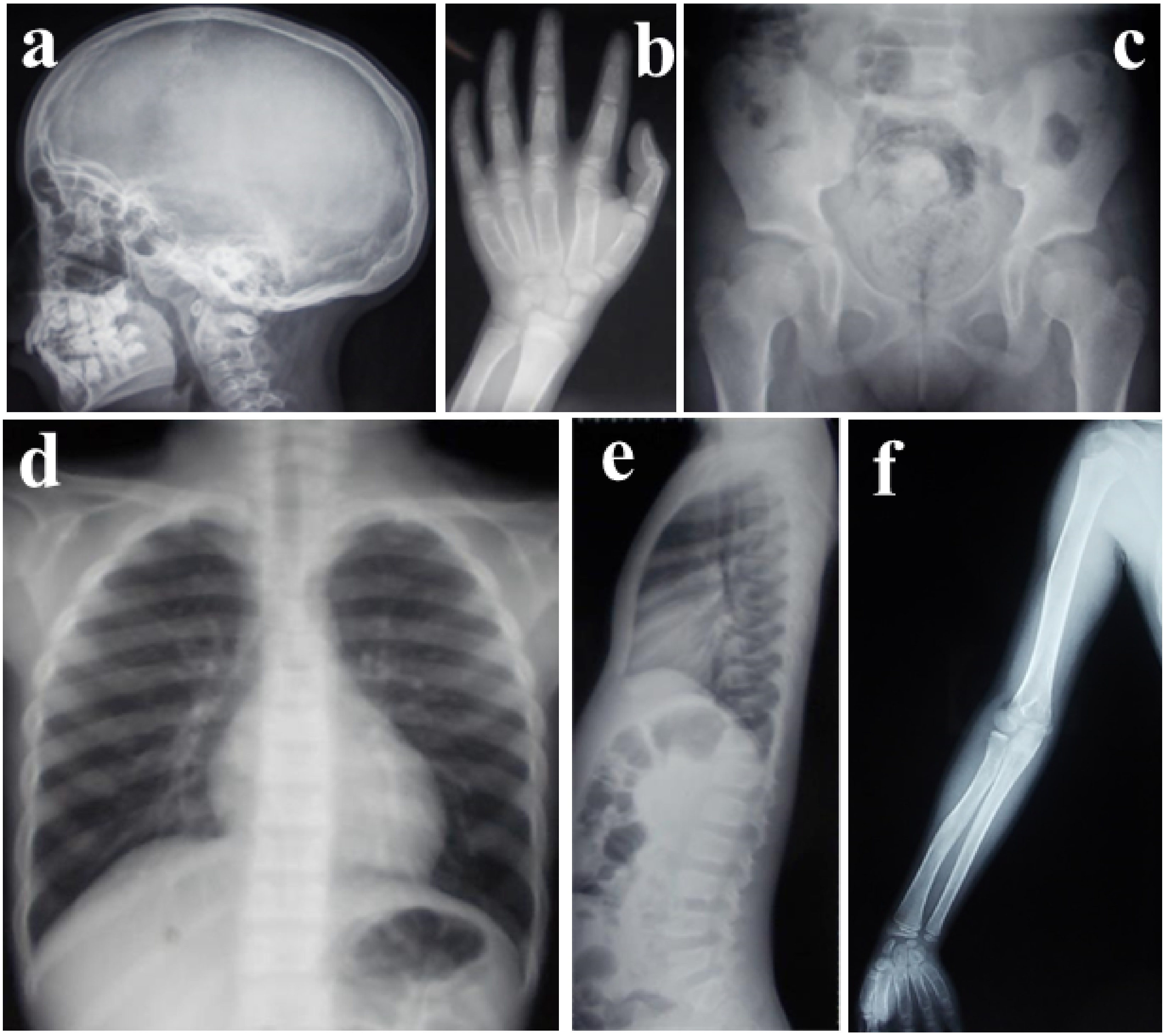

Supplementary Fig. 19: Radiographs of Individual B-III-18 at age 8.
a, Thick calvaria. **b,** Broad and short tubular bones of hands. **c,** Widening of pubis and ischia, and broadening of the femoral neck. **d,** Broad ribs. **e,** Mild platyspondyly and broad ribs. **f,** The long tubular bones of the upper limbs are normal.

Supplementary Fig. 20

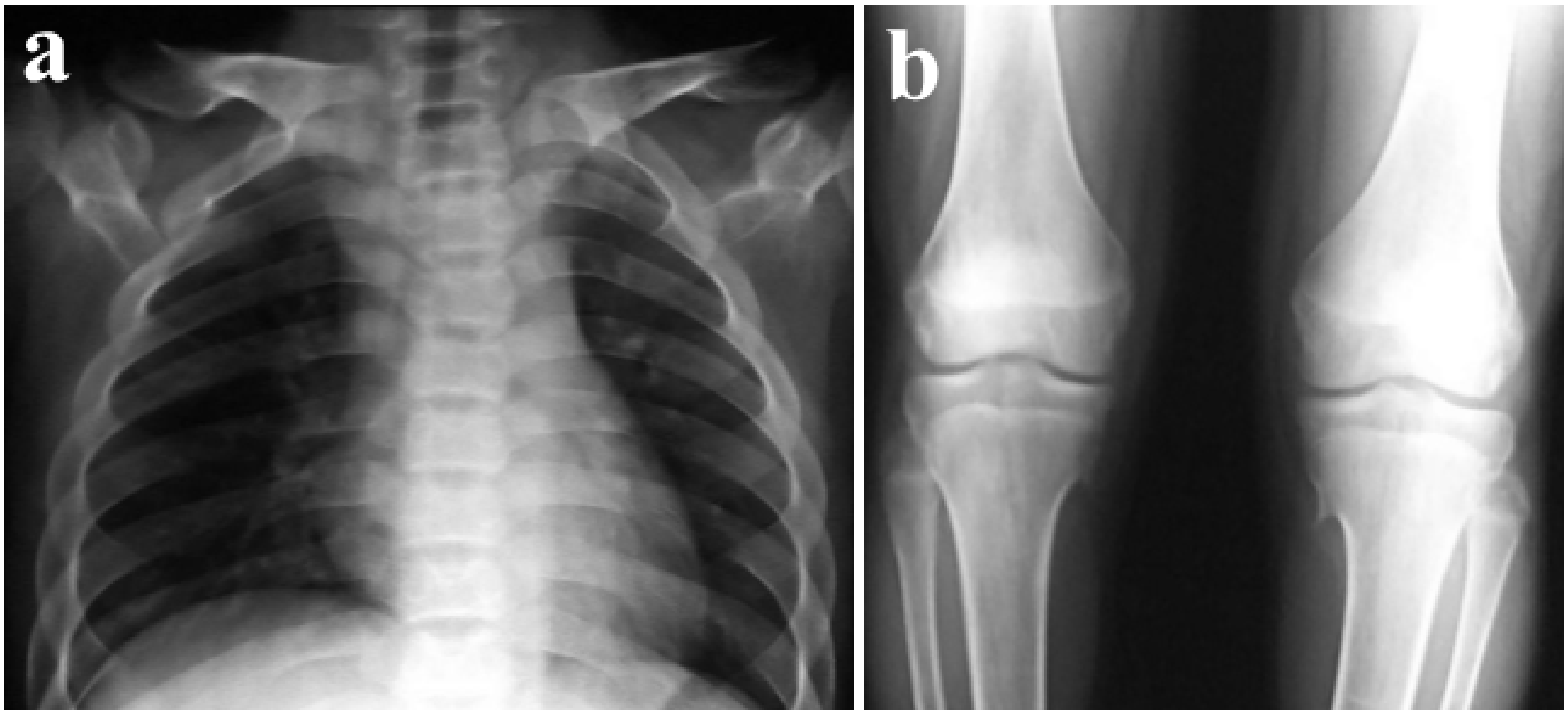

Supplementary Fig. 20: Radiographs of Individual C-II-1 at age 15.
a, Mild platyspondyly and broad ribs. **b,** Mild metaphyseal dysplasia of knee joints.

Supplementary Fig. 21

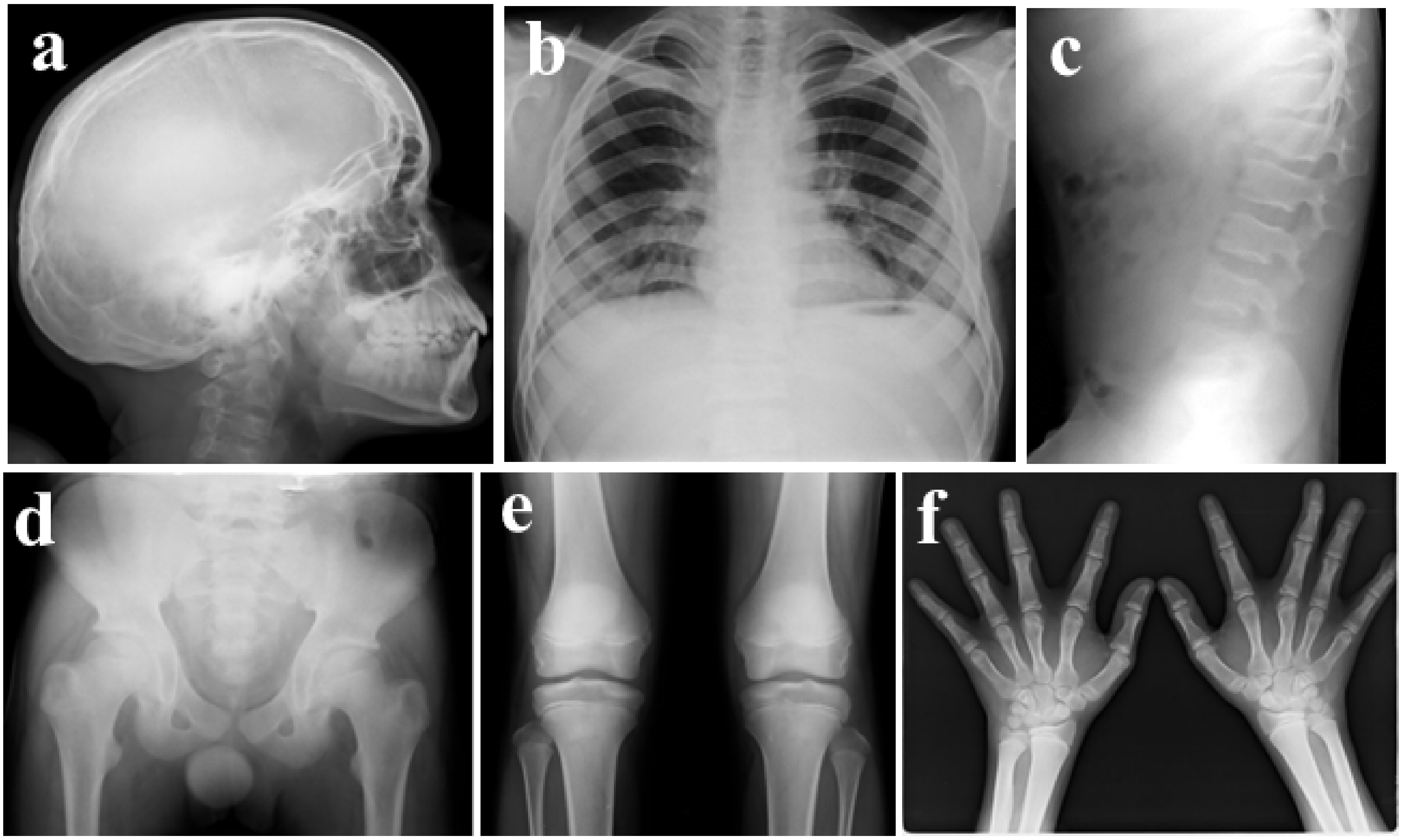

Supplementary Fig. 21: Radiographs of Individual D-III-4 at age 17.
a, Thick calvaria. **b,** Broad ribs. **c,** Mild platyspondyly and broad ribs. **d,** Widening of pubis and ischia, broadening of the femoral neck. **e,** Mild metaphyseal dysplasia of the knee joints **f,** The tubular bones of hands are broad and short.

Supplementary Fig. 22

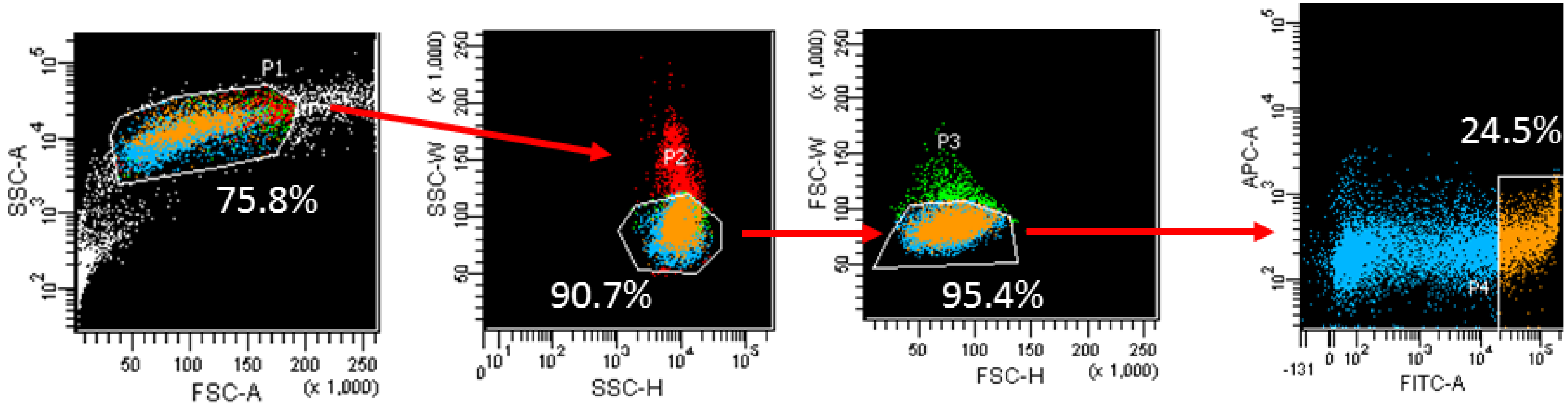

Supplementary Fig. 22: Gating strategy for sorting transfected Hela cells.
The pair of pX330 plasmids with the highest efficiency of editing *TMEM53* genomic sequence were selected and co-transfected with pEGFP-N1 vector. pEGFP-N1 expresses EGFP, which is used as a fluorescent marker (FITC) to sort pX330-transfected cells by a flow cytometry.

Supplementary Table 1. Throughput of the whole exome sequencing

Subject	Read		Coverage (%)	
	Total (Gb)	Mean depth	≥ 10 read	≥ 20 read
A-V-1	2.5	×75.6	96.0	91.7
A-V-2	2.5	×74.6	96.0	91.5
B-III-18	1.7	×51.5	64.1	85.3
C-II-1	3.4	×74.8	96.8	91.0
D-III-4	6.2	×136.7	99.0	97.6

Calculated based on the entire coding regions or the entire genomic sequence of human genome browser, hg19.

Supplementary Table 2. *In silico* prediction of pathogenicity of the *TMEM53* splice-site variant, c.62-5_62-3delTTC by online programs

Sequence	Score of splice acceptor site					
	NNSplice	ASSP	HSF	MaxEntScan*		
				MAXENT	MM	WMM
Wild type	0.65	5.040	86.78	7.11	6.18	4.74
c.62-5_62-3delTTC	0.13	3.009	75.67	2.46	1.31	1.47
Variation (%)	-80	-40	-13	-65	-79	-69

NNSplice, Splice Site Prediction by Neural Network. ASSP, Alternative Splice Site Predictor. HSF, Human Splicing Finder.

* MaxEntScan includes three different prediction models: MAXENT, Maximum Entropy Model; MM, First-order Markov Model; WMM, Weight Matrix Model.

Supplementary table 3. List of the primers used in this study

Primer name	Sequence (5’-3’)
Expression plasmid construction	
TMEM53 T1T3 F1	TCCGAATTCTATGGCCTCGGCAGAGC
TMEM53 T1T2T3 R1	CGCAAGCTTTCAGCAGCGGACGCAG
TMEM53 T2 F1	TCCGAATTCTATGGTCTTCTTCTCCGAGTCAC
TMEM53 T1mut F1	CATGGTCTTCTTCTCCGAGTCAC
TMEM53 T1mut R1	CATGTGCCACGGGGCT
TMEM53 T1mut F2	AAGCTTGCGGCCGCAC
TMEM53 T1mut R2	TCAAAGAGCAGCTCGAGCAG
TMEM53 T2mut F1	TCCGAATTCTATGCATGGTCTTCTTCTCCGAGTCACTGG
TMEM53 T2mut R1	CGCAAGCTTTCAAAGAGCAGCTCGAGCAG
TMEM53 T3mut F1	AAGCTGCTCGAGCTGCTCTTT
TMEM53 T3mut R1	TCTGGCTCCAGCAGGGC
TMEM53 T3mut F2	AAGCTTGCGGCCGCACA
TMEM53 T3mut R2	TCAAAGAGCAGCTCGAGCAGC
Sanger sequencing	
TMEM53 EX2F	CAGCCGGAGTGCTGCTGA
TMEM53 EX2R	TCCCAGTCCTGCCTCAAAGGT
TMEM53 EX3F	AGCAGGACCCAGAGCATTGCT
TMEM53 EX3R	GTACAGCATGACGCCACCGTT
RT-PCR	
TMEM53 5UTR RT FP	TCCGGCTGGAGACCCGT
TMEM53 EX3 RP	GTACAGCATGACGCCACCGTT
pTriEx4_tag_rtpcr_f1	ATGGCACACCATCACCACC

pTriEx4_tag_rtpcr_r1	GAATTCGGATCCACGAGCTCAG
Genome editing for cells	
TMEM53 e3 g1 f	CACCGGCACCTGTGTCTGTGGGCAG
TMEM53 e3 g1 r	AAACCTGCCCACAGACACAGGTGCC
TMEM53 e3 g2 f	CACCGGCTTACCACAGAATCCTAAG
TMEM53 e3 g2 r	AAACCTTAGGATTCTGTGGTAAGCC
TMEM53 e3 g3 f	CACCGCAGAGCATTGCTGACGCTGT
TMEM53 e3 g3 r	AAACACAGCGTCAGCAATGCTCTGC
TMEM53 e3 g4 f	CACCGAAGAGTGCCCGACAGATTGC
TMEM53 e3 g4 r	AAACGCAATCTGTCGGGCACTCTTC
TMEM53 e3 g5 f	CACCGGGGCAGGGAAGGTATACTTA
TMEM53 e3 g5 r	AAACTAAGTATACCTTCCCTGCCCC
TMEM53 e3 g6 f	CACCGGCAAAGTCCCAAAGGGCTAC
TMEM53 e3 g6 r	AAACGTAGCCCTTTGGGACTTTGCC
TMEM53 crisprE3del F	CCAAATGTGCAAGGCCTAGGATG
TMEM53 crisprE3del R	TCCATTTGTCAACCTCTACAGCCTG
**Genome editing for mice**	
Oligo tet1-site1-F	CCAAGCTTTAATACGACTCACTATAGGCAACCTGATAGGGGCCCTGGTTTTAGAGCTAGA AATAGCAAGT
gRNA-F101	CCAAGCTTTAATACGACTCAC
gRNA-R101	CCTCTAGAAAAGCACCGACTC
gRNA-R-XbaI	CCTCTAGAAAAGCACCGACTCGGTGCCACTTTTTCAAGTTGATAACGGACTAGCCTTATTT TAACTTGCTATTTCTAGCTCTAAAAC
Tmem53 seq f	GGCTTCTTCGGCTAACCTGT
Tmem53 seq r	AAGAGGGCAGTGAATGGAGC
RT-qPCR	

TMEM53 qPCR f1	CCAAGTACAGTGCCATCTACCAC
TMEM53 qPCR r1	GGATACCCAGTGACTCGGAGAA
Tmem53 qPCR f1	GCAACAGCCTGTGGTGATTCTC
Tmem53 qPCR r1	CGTGTATCGGATCACAATGCAGC
Runx2 qPCR f1	CCTCTGACTTCTGCCTCTGG
Runx2 qPCR r1	ATGAAATGCTTGGGAACTGC
Sp7 qPCR f1	GATGGCGTCCTCTCTGCTT
Sp7 qPCR r1	AGCGTATGGCTTCTTTGTGC
Alpl qPCR f1	GCTGATCATTCCCACGTTTT
Alpl qPCR r1	CTGGGCCTGGTAGTTGTTGT
Bglap qPCR f1	AAGCAGGAGGGCAATAAGGT
Bglap qPCR r1	TTTGTAGGCGGTCTTCAAGC
Ibsp qPCR f1	GAGACGGCGATAGTTCCGAAGAG
Ibsp qPCR r1	AGCTGTTACACCCGAGAGTGTGG
Gapdh qPCR f1	ACCACAGTCCATGCCATCAC
Gapdh qPCR r1	TCCACCACCCTGTTGCTGTA
Col2a1 qPCR f1	GCCAAGACCTGAAACTCTGC
Col2a1 qPCR r1	GCCATAGCTGAAGTGGAAGC
Acan qPCR f1	CCAAACCAGCCTGACAACTT
Acan qPCR r1	TCTAGCATGCTCCACCACTG
ISH probe synthesis	
Tmem53 AS f1	CCGCTCGAGTAAAGGTTGAAGGGAGGTGTCG
Tmem53 AS r1	CGCGGATCCAGACCCACCAGCGCTTC
Tmem53 S f1	CCGCTCGAGAGACCCACCAGCGCTTC
Tmem53 S r1	CGCGGATCCTAAAGGTTGAAGGGAGGTGTCG
