# Supplementary material for: Deficiency of TMEM53 causes a previously unknown sclerosing bone disorder by dysregulation of BMP-SMAD signaling
Source: Nat Commun. 2021 Apr 6;12:2046. doi: 10.1038/s41467-021-22340-8 (PMC8024261; doi:10.1038/s41467-021-22340-8)
Supplement: Supplementary file 2 — Reporting Summary [file 41467_2021_22340_MOESM2_ESM.pdf]

nature research

Corresponding author(s): Long Guo, Katta M. Girisha, Shiro Ikegawa

Last updated by author(s): Feb 1, 2021

Reporting Summary

Nature Research wishes to improve the reproducibility of the work that we publish. This form provides structure for consistency and transparency in reporting. For further information on Nature Research policies, see our Editorial Policies and the Editorial Policy Checklist.

Statistics

For all statistical analyses, confirm that the following items are present in the figure legend, table legend, main text, or Methods section.

n/a	Confirmed	
☐	☒	The exact sample size (n) for each experimental group/condition, given as a discrete number and unit of measurement
☐	☒	A statement on whether measurements were taken from distinct samples or whether the same sample was measured repeatedly
☐	☒	The statistical test(s) used AND whether they are one- or two-sided<br>*Only common tests should be described solely by name; describe more complex techniques in the Methods section.*
☐	☒	A description of all covariates tested
☐	☒	A description of any assumptions or corrections, such as tests of normality and adjustment for multiple comparisons
☐	☒	A full description of the statistical parameters including central tendency (e.g. means) or other basic estimates (e.g. regression coefficient) AND variation (e.g. standard deviation) or associated estimates of uncertainty (e.g. confidence intervals)
☐	☒	For null hypothesis testing, the test statistic (e.g. F, t, r) with confidence intervals, effect sizes, degrees of freedom and P value noted<br>*Give P values as exact values whenever suitable.*
☒	☐	For Bayesian analysis, information on the choice of priors and Markov chain Monte Carlo settings
☒	☐	For hierarchical and complex designs, identification of the appropriate level for tests and full reporting of outcomes
☒	☐	Estimates of effect sizes (e.g. Cohen's d, Pearson's r), indicating how they were calculated

Our web collection on statistics for biologists contains articles on many of the points above.

Software and code

Policy information about availability of computer code

Data collection	HiSeq 2000 Sequencer (Illumina), NextSeq500 Sequencer (Illumina), NovaSeq 6000 sequencer (Illumina), ABI 3730 DNA analyzer (Life Technologies), Nikon A1Rsi microscope (Nikon), MVX10 microscope (Olympus), Scan Xmate-L090 (Comscan), X-ray TRS-1005 (SOFRON), StepOnePlus Real-Time PCR system (Applied Biosystems).
Data analysis	CASAVA1.6.0 or 1.8.2 (Illumina), Novoalign (ver. 3.02.04), BWA-MEM (ver. 0.7.15), Picard (v.1.128), Genome Analysis Toolkit (GATK v2.7-4), ANNOVAR (version 2014 July 14), Sequencher V.4.7 (Gene Codes), Genetyx Ver.12, FASTX-toolkit (version 0.0.14_1), Tophat (2.1.1_6), Cufflinks (v2.1.1), Cuffdiff (v2.1.1), RStudio (ver. 1.0.153), GraphPad Prism 8.0, DAVID 6.8, Avizo 6.3, and Fiji (Java6-20170530).

For manuscripts utilizing custom algorithms or software that are central to the research but not yet described in published literature, software must be made available to editors and reviewers. We strongly encourage code deposition in a community repository (e.g. GitHub). See the Nature Research guidelines for submitting code & software for further information.

Data

Policy information about availability of data

All manuscripts must include a data availability statement. This statement should provide the following information, where applicable:

- Accession codes, unique identifiers, or web links for publicly available datasets
- A list of figures that have associated raw data
- A description of any restrictions on data availability

The human variant data have been deposited in NCBI ClinVar with the accession codes “SCV001446315 [https://www.ncbi.nlm.nih.gov/clinvar/variation/986737/]” and “SCV001446316 [https://www.ncbi.nlm.nih.gov/clinvar/variation/986738/]”. The variants were evaluated by using the databases: GeneBank (https://www.ncbi.nlm.nih.gov/genbank/), dbSNP (http://www.ncbi.nlm.nih.gov/projects/SNP/), ExAC (http://exac.broadinstitute.org/), gnomAD (https://gnomad.broadinstitute.org/), HGMD (https://portal.biobase-international.com/hgmd/pro/start.php), NNSPLICE (http://www.fruitfly.org/seq_tools/splice.html),

ASSP (http://wangcomputing.com/assp/), HSF (http://www.umd.be/HSF/), and MaxEntScan (http://genes.mit.edu/burgelab/maxent/Xmaxentscan_scoreseq.html). Homozygosity mapping was performed by HomozygosityMapper (http://www.homozygositymapper.org/). The mouse RNA-seq data have been deposited in NCBI GEO with the accession code "GSE161193 [https://www.ncbi.nlm.nih.gov/geo/query/acc.cgi?acc=GSE161193]". Source data underlying Figs. 2c, 2f, 3c, 3e, 3g, 3i, 3k, 3m, 4b, 4c, 4d, 4e, 5a, 5c-f, 5h-j, 5l, and Supplementary Figs. 2, 6, 8b, 9a-b, 11b, 12e, 13, 14, 16c, 16d-f are provided as a Source Data file. The patients' genome data obtained by whole-exome sequencing are not publicly available because study participants did not give full consent for releasing data publicly. These data can be accessed under the condition that a joint research plan is made by the researchers and approved by the ethics committees. All the other data supporting the findings of this study are included within the article or the supplementary information.

Field-specific reporting

Please select the one below that is the best fit for your research. If you are not sure, read the appropriate sections before making your selection.

☒ Life sciences ☐ Behavioural & social sciences ☐ Ecological, evolutionary & environmental sciences

For a reference copy of the document with all sections, see nature.com/documents/nr-reporting-summary-flat.pdf

Life sciences study design

All studies must disclose on these points even when the disclosure is negative.

Sample size	Specific samples sizes are indicated in figure legends. In animal study, the sample size was estimated based on our prior experience of performing similar sets of experiments (Takimoto et al. Development 2015 142: 787-796). In molecular biology experiments, n>=3 was chosen because it is sufficient to conduct statistical tests to generate p-values to determine if results are significant.
Data exclusions	No data were excluded.
Replication	Human DNA samples were sequenced using whole-exome sequencing once, and the mutation was confirmed using Sanger sequencing in three independent experiments. All the attempts at replication were successful. The RNA-seq for mouse calvaria was performed once. In molecular biology experiments, we independently repeated the experiments at least twice, and all attempts to reproduce the results were successful.
Randomization	Randomization was not performed. The mice were selected based on the genotype we needed. Sample randomization is not applicable for cell culture experiments where batches of homogeneous cultures can be tested in parallel.
Blinding	Blinding was performed for micro-CT analysis. For other experiments, the investigators were not blinded to group allocation during data collection and/or analysis.

Reporting for specific materials, systems and methods

We require information from authors about some types of materials, experimental systems and methods used in many studies. Here, indicate whether each material, system or method listed is relevant to your study. If you are not sure if a list item applies to your research, read the appropriate section before selecting a response.

Materials & experimental systems

n/a	Involved in the study	
☐	☒	Antibodies
☐	☒	Eukaryotic cell lines
☒	☐	Palaeontology and archaeology
☐	☒	Animals and other organisms
☐	☒	Human research participants
☒	☐	Clinical data
☒	☐	Dual use research of concern

Methods

n/a	Involved in the study	
☒	☐	ChIP-seq
☐	☒	Flow cytometry
☒	☐	MRI-based neuroimaging

Antibodies

Antibodies used	Anti-Smad1 (WB, 1:1,000 dilution, #9743, Cell signaling technology), anti-phospho-Smad1/5/9 (WB, 1:1,000 dilution, #13820, Cell signaling technology), anti-β-actin-HRP (WB, 1:10,000 dilution, PM053-7, MBL, Japan), anti-Lamin A (WB, 1:1,000 dilution, ab226198, Abcam), anti-β-tubulin (WB, 1:1,000 dilution, H-235, Santa Cruz), anti-rabbit IgG–peroxidase (WB, 1:20,000 dilution, A0545, Sigma-Aldrich), and S-protein HRP conjugate (WB, 1:5,000 dilution, 69047, Novagen). Anti-S-tag dylight 488 (ICC, 1:500 dilution, ab117509, Abcam), anti-phospho-Smad1/5/9 antibody (ICC, 1:100 dilution, #13820, Cell signaling technology), anti-lamin A (ICC, 1:1,000 dilution, #86846, Cell signaling technology), anti-rabbit IgG-fluor 546 (ICC, 1:250 dilution, A11035, Invitrogen), and anti-mouse IgG-fluor 546 (ICC, 1:500 dilution, A11030, Invitrogen).
Validation	All antibodies are well-validated by the manufacturer and are widely used in the scientific community. The validation information is available by: https://en.cellsignal.jp/products/primary-antibodies/smad1-antibody/9743; https://en.cellsignal.jp/products/primary-antibodies/phospho-smad1-ser463-465-smad5-ser463-465-smad9-ser465-467-d5b10-rabbit-mab/13820; https://ruo.mbl.co.jp/bio/

dtl/A/?pcd=PM053-7; https://www.abcam.com/lamin-a-antibody-ab226198.html; https://www.scbt.com/p/beta-tubulin-antibody-h-235; https://www.sigmaaldrich.com/catalog/product/sigma/a0545?lang=en&region=US; https://us.vwr.com/assetsvc/asset/en_US/id/10908454/contents; https://www.abcam.com/dylight-488-s-tag-antibody-ab117509.html; https://en.cellsignal.jp/products/primary-antibodies/phospho-smad1-ser463-465-smad5-ser463-465-smad9-ser465-467-d5b10-rabbit-mab/13820; https://www.cellsignal.com/products/primary-antibodies/lamin-a-133a2-mouse-mab/86846; https://www.thermofisher.com/antibody/product/Goat-anti-Rabbit-IgG-H-L-Highly-Cross-Adsorbed-Secondary-Antibody-Polyclonal/A-11035; https://www.thermofisher.com/antibody/product/Goat-anti-Mouse-IgG-H-L-Highly-Cross-Adsorbed-Secondary-Antibody-Polyclonal/A-11030.

Eukaryotic cell lines

Policy information about cell lines

Cell line source(s)	MG-63, MC3T3-E1 , ATDC5 and Hela cells were obtained from RIKEN BioResource Research Center. TMEM53 knock-out Hela cell lines were generated on the basis of wild type Hela cells by genome editing, which causes a large deletion covering exon 3. The information about the TMEM53 knock-out Hela cells is shown in Supplementary Fig. 12.
Authentication	Cell lines were not authenticated.
Mycoplasma contamination	All cell lines were tested negative for mycoplasma contamination.
Commonly misidentified lines (See ICLAC register)	No commonly misidentified cell lines were used

Animals and other organisms

Policy information about studies involving animals; ARRIVE guidelines recommended for reporting animal research

Laboratory animals	Tmem53 mutant mice were generated and kept in the B6N background. Mice from E16.5 to P113 (E16.5, P1, P2, P3, P4, P7, P24, P93, P113) were used for the study. The age and sex were specified in the figure legends. The mice were housed at a standard 12-h light/12-h dark cycle. The ambient temperatures is 21-25°C with 40-60% humidity.
Wild animals	This study did not involve wild anima
Field-collected samples	This study did not involve samples collected from the field.
Ethics oversight	All animal experimental protocols were approved by the Animal Experiments Committee of the RIKEN Center for Integrative Medical Sciences and conformed to institutional guidelines for the study of vertebrates. All animal experiments were carried out in accordance with the in-house guidelines for the care and use of laboratory animals of the RIKEN, Yokohama Institute, Japan.

Note that full information on the approval of the study protocol must also be provided in the manuscript.

Human research participants

Policy information about studies involving human research participants

Population characteristics	All five patients and their family members were Indians from different geographic areas. The age, gender, genotypic information, diagnosis and treatment were specified in the section of Methods/Patient. Specifically, the patient A-V-1 is a 19-year-old man. His past and current diagnosis is unknown sclerosing bone disorderand and impaired vision. Right frontal orbitotomy was done for deterioration of vision. The patient A-V-2 is a 12-year-old girl. Her past and current diagnosis is unknown sclerosing bone disorder and impaired vision. No specific treatment was performed. The patient B-III-18 is a 8-year-old girl. Her past and current diagnosis is unknown sclerosing bone disorder and impaired vision. No specific treatment was performed. The patient C-II-1 is a 15-year-old boy. His past and current diagnosis is unknown sclerosing bone disorder and impaired vision. No specific treatment was performed. The patient D-III-4 is a 17-year-old man. His past and current diagnosis is unknown sclerosing bone disorder, impaired vision, syndactyly of the left third and fourth fingers. No specific treatment was performed.
Recruitment	The patients with an extraordinary sclerosing bone disorder (SBD) were found in routine clinical practice and were initially evaluated by experienced human geneticists and clinicians (K.M.G., S.L.B., K.G.), independently. Then, the clinical phenotypes were sent to two experts in human skeletal dysplasias (G.N. and S.I.) via Japan Skeletal Dysplasia Consortium (http://www2.riken.jp/lab/OA-team/JSDC/) for a detailed evaluation. These independent clinical analyses identified four unrelated Indian families affected by the same unknown type of SBD. We therefore consider that there is no clinical selection bias in our study.
Ethics oversight	The study was approved by the ethical committee of RIKEN and participating institutions.

Note that full information on the approval of the study protocol must also be provided in the manuscript.

Flow Cytometry

Plots

Confirm that:

- [x] The axis labels state the marker and fluorochrome used (e.g. CD4-FITC).
- [x] The axis scales are clearly visible. Include numbers along axes only for bottom left plot of group (a 'group' is an analysis of identical markers).
- [x] All plots are contour plots with outliers or pseudocolor plots.
- [x] A numerical value for number of cells or percentage (with statistics) is provided.

Methodology

Sample preparation	Hela cells were transfected by pX330 and pEGFP-N1 plasmids.
Instrument	FACSAria (BD Biosciences)
Software	FACSDiva Ver. 6.1.2
Cell population abundance	About 140000 cells.
Gating strategy	Gating strategies are shown in Supplementary Fig. 22. Briefly, FITC-positive cells were gated by gating on SSC-A and FSC-A, subsequently SSC-H and SSC-W, FSC-H and FSC-W, finally FITC-A and APC-A.

- [x] Tick this box to confirm that a figure exemplifying the gating strategy is provided in the Supplementary Information.
